# Straglr: discovering and genotyping tandem repeat expansions using whole genome long-read sequences

**DOI:** 10.1186/s13059-021-02447-3

**Published:** 2021-08-13

**Authors:** Readman Chiu, Indhu-Shree Rajan-Babu, Jan M. Friedman, Inanc Birol

**Affiliations:** 1 Canada’s Michael Smith Genome Sciences Centre, BC Cancer, Vancouver, BC V5Z 4S6 Canada; 2 Department of Medical Genetics, University of British Columbia, Vancouver, BC V6T 1Z3 Canada; 3 BC Children’s Hospital Research Institute, Vancouver, BC V5Z 4H4 Canada; 4 Department of Medical and Molecular Genetics, King’s College London, Strand, London, WC2R 2LS UK

## Abstract

Tandem repeat (TR) expansion is the underlying cause of over 40 neurological disorders. Long-read sequencing offers an exciting avenue over conventional technologies for detecting TR expansions. Here, we present Straglr, a robust software tool for both targeted genotyping and novel expansion detection from long-read alignments. We benchmark Straglr using various simulations, targeted genotyping data of cell lines carrying expansions of known diseases, and whole genome sequencing data with chromosome-scale assembly. Our results suggest that Straglr may be useful for investigating disease-associated TR expansions using long-read sequencing.

**Supplementary Information:**

The online version contains supplementary material available at 10.1186/s13059-021-02447-3.

## Background

Long-read or third-generation DNA sequencing is increasingly employed in genomic applications such as de novo assembly, structural variant detection, transcriptome profiling, and metagenomics [1]. The Oxford Nanopore Technologies (Nanopore) and PacBio Single Molecule, Real-Time (SMRT) long-read sequencing platforms produce reads with sizes ranging from tens to hundreds of kilobases (kb) [2, 3], enabling the characterization of long-range structural genomic information that is unattainable by short reads. While a major limitation of long-read technologies has been their higher per nucleotide error rate (10–15%) [4], this constraint has been mitigated by improvements in the sequencing technologies and accompanying processing algorithms [3]. As these technologies have developed, their costs have declined and their throughput has increased. Long-read sequencing is now widely applied in whole genome sequencing (WGS) efforts [1, 5].

Short tandem repeats (STRs) are genomic segments typically composed of 2–6 base pair (bp) units repeated in tandem and in the same orientation. STRs comprise about 3% of the human genome and are highly polymorphic. Lengths of certain STRs play biological roles. There are human diseases caused by STR expansions, where STR lengths beyond a certain threshold are known to be pathogenic [6]. The threshold length differs by STR locus and disease, and longer repeats can cause more severe and earlier onset of clinical symptoms in patients with some repeat expansion disorders [7]. Additionally, repeat size may also be strongly associated with heterogeneity in phenotype manifestations, even among patients with the same disease [8]. Trinucleotides are the repeat unit, or repeat motif, that has predominantly been found in diseases to date, but more recently, expansions in longer and more complex tandem repeats (TRs), some with repeat units > 6 bp in size (called variable number tandem repeats, VNTRs), have also been found to be pathogenic [9–12].

Conventionally, clinical STR genotyping is performed by polymerase chain reaction (PCR) or Southern blot assays [13]. Both methods can only be used to analyze one or a few target STR loci at a time, which makes second-generation (short-read) DNA sequencing an attractive alternative for analyzing exome- or genome-wide STRs with a single test. However, the detectable size range of STR expansions is limited by the read length of the sequencing technology, typically in the range of 120–150 bp for second-generation sequencing. For repeats longer than the read length, sequencing reads composed predominantly or entirely of repeat sequences cannot be mapped reliably, if at all, to the reference genome [14]. Algorithms have recently been developed to estimate larger-than-read-length expansion sizes with sophisticated statistical modeling [15–17]. The success of these approaches depends on accurate identification of partially mapped paired-end reads and unmapped or mis-mapped full-repeat reads originated from the STR loci in question. Accurately aligning such reads is particularly challenging for expansions that exceed sequencing fragment length, typically 350–500 bp. Hence, under-estimation of repeat sizes often occurs in such cases [18]. Moreover, traditional short-read sequencing libraries prepared with PCR amplification exhibit GC-bias, resulting in under-representation of sequence reads originated from STRs with high GC content. This presents a major problem for STR expansion genotyping as some of the known diseases arise from repeats comprised entirely of guanine and cytosine. While PCR-free sequencing libraries improve coverage of GC-rich regions, they may not entirely provide a solution for reliable STR genotyping using short reads due to the caveats listed above.

Long-read sequencing offers an opportunity to remedy these problems, especially for STRs that are kb, and sometime tens of kb, in length [19]. Library preparation for SMRT or Nanopore long-read sequencing does not require PCR amplification and individual DNA molecules are sequenced without amplification in both technologies. Therefore, long reads are not subject to GC bias as much as short-reads are [20, 21]. To date, a number of studies have demonstrated the potential utility of long-read sequencing for genotyping expanded STRs in cell lines or patient samples [22, 23]. Advancements continue to be made in this direction, the latest example being the use of the CRISPR-Cas9 system for target enrichment to achieve highly accurate STR length genotypes [24, 25].

More than 40 diseases have been found to be caused by STR expansions [8]. Repeat expansions may also be the mechanism underlying other rare diseases that are currently unexplained [26–31]. Efficiently searching for expansions anywhere in the genome would greatly facilitate the identification of novel disease-associated STR loci, but performing a genome-wide search for such expansions with existing long-read genotyping software necessitates interrogating hundreds of thousands of annotated loci (e.g., the UCSC simple repeats track), a time- and resource-intensive process.

We developed Straglr, a new software tool that scans the entire genome for potential TR expansions by first extracting insertions composed of TRs and then genotyping the identified “expanded” loci. This approach not only spares the time and computing resources required for genotyping thousands of non-expanded TR loci but also enables the discovery of expansions at previously unannotated loci. Herein we have demonstrated Straglr’s performance in genotyping STR expansions using both simulated and real long-read data and evaluated its results of a genome scan against a diploid genome assembly.

## Results

### Simulated data

From the human reference genome (hg38), we generated three genomic sequences, each modified at 17 known STR disease loci (Table 1) on different chromosomes to contain alleles of a fixed size of 100, 500, or 4000 bp. We used NanoSim [32] to stimulate Nanopore reads based on the accompanying NA12878 Guppy flip-flop model [33]. We combined an equivalent number of Nanopore reads from the modified and unmodified reference sequences to create simulated genomes with approximately 30X coverage composed of one normal and one expanded STR allele at the 17 disease loci. These heterozygous samples were used to evaluate Straglr and two other long-read STR genotyping tools: tandem-genotypes (v1.4.0) [23] and RepeatHMM (v1.0) [22].

**Table 1:** Known disease loci used for simulation

Chromosome	Start	End	Gene	Motif
chr2	176,093,057	176,093,099	*HOXD13*	GGC
chr3	63,912,685	63,912,715	*ATXN7*	CAG
chr4	3,074,876	3,074,933	*HTT*	CAG
chr5	146,878,728	146,878,758	*PPP2R2B*	CTG
chr6	16,327,635	16,327,722	*ATXN1*	CTG
chr7	27,199,924	27,199,966	*HOXA13*	CGC
chr9	69,037,286	69,037,304	*FXN*	GAA
chr11	119,206,289	119,206,322	*CBL*	CGG
chr12	111,598,950	111,599,019	*ATXN2*	CTG
chr13	70,139,383	70,139,428	*ATXN8*	CTG
chr14	92,071,010	92,071,034	*ATXN3*	CTG
chr16	87,604,287	87,604,329	*JPH3*	CTG
chr19	45,770,204	45,770,264	*DMPK*	CAG
chr20	2,652,733	2,652,775	*NOP56*	GGCCTG
chr21	43,776,443	43,776,479	*CSTB*	CGCGGGGCGGGG
chr22	45,795,354	45,795,424	*ATXN10*	ATTCT
chrX	147,912,050	147,912,110	*FMR1*	CGG

**Fig. 1:**
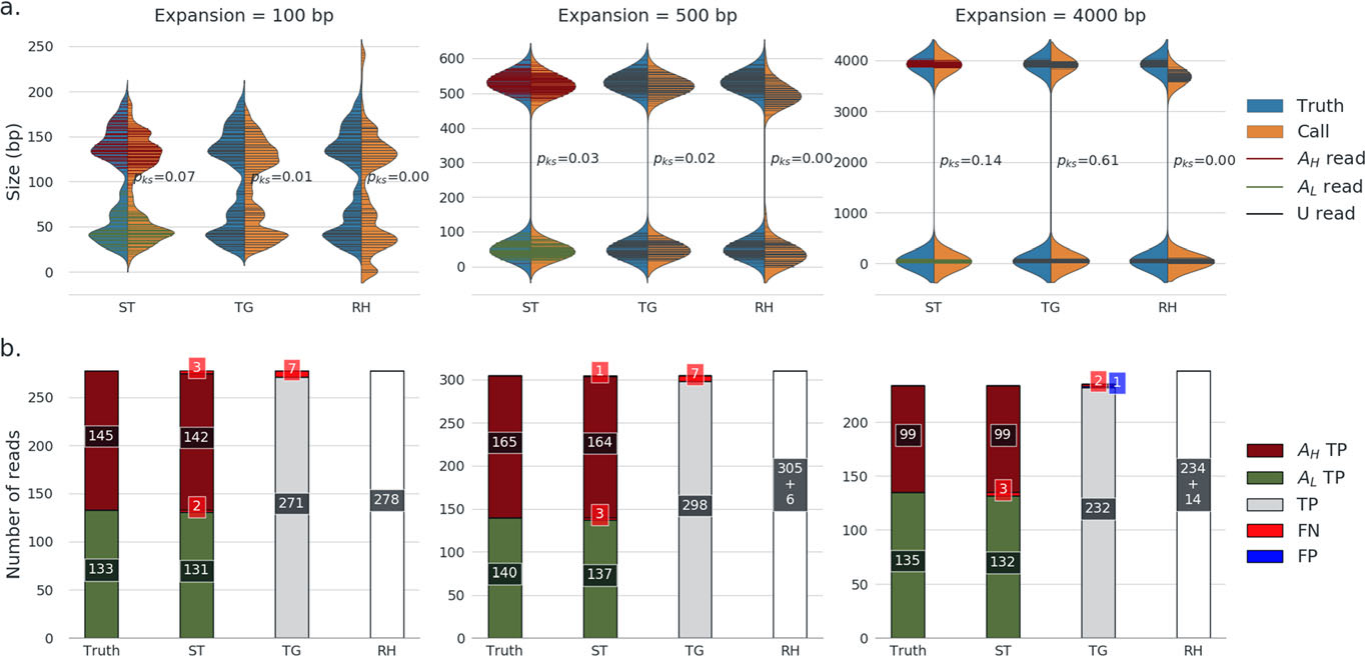
Genotyping benchmark (simulated data): repeat capture. **a** Repeat size distribution of genotyping results from Straglr (ST), tandem-genotypes (TG) and RepeatHMM (RH) compared against real sizes (Truth) in simulated samples. Each sample is composed of 17 heterozygous loci (Table 1) with a reference and expanded alleles. Violin plot of each tool (orange, right) is juxtaposed with violin plot of the real distribution (blue, left) in each of the nine samples with different expansion sizes. Horizontal lines within the violin plot indicate the actual repeat sizes (y-positions) and relative frequencies (widths) detected. Red lines indicate sizes classified (ST) or generated (Truth) as the expanded allele (A_H_), green the reference (A_L_) allele, and black unclassified. P_KS_ indicates the *p* value from a KS test comparing the tool’s estimated and truth repeat size distributions. **b** True-positive (TP), false-positive (FP), and false-negative (FN) histograms in each of the nine experiments. Classifications are separated for expanded (dark red) and reference (green) alleles in ST based on the reported genotypes. No classification is possible with RH as supporting read identities were not revealed. Numbers in RH just indicate the total number of Truths reads plus the difference detected; e.g., 305 + 6 indicate 311 reads in total were detected by RH, 6 more than the total truth

To evaluate the performance of each tool in genotyping STRs from long reads, we first compared repeat size distributions determined from individual reads against the actual size distributions (Fig. 1a). To determine the actual repeat size distribution, we first identified the subset of simulated reads that spanned the 17 loci based on coordinate and size information encapsulated in the NanoSim read names. The actual sizes of the embedded STRs were calculated from alignment of sequences flanking the target loci to reads originated from the corresponding locus (see the “Methods” section). As expected, repeat sizes reported by all three tools exhibited bi-modal distributions in all heterozygous samples. We performed a two-sample Kolmogorov-Smirnov (KS) test using a *p* value of < 0.05 to indicate a significant difference between the actual and reported repeat size distribution for all tools in all samples. Straglr showed a *p* value smaller than the cutoff in only one sample (expansion 500 bp), whereas tandem-genotypes showed *p* values smaller than the cutoff in two samples (expansions 100 and 500 bp). RepeatHMM exhibited a statistically different (*p* < 0.01) repeat size distribution from the actual one at all expansions, mostly likely due to its under-sizing of the larger alleles, which was visually observable in all cases.

We then compared the numbers and identities of target-repeat harboring reads reported by the three tools against the ground truth (Fig. 1b). As RepeatHMM does not reveal the identities of support reads used in genotyping, only Straglr and tandem-genotypes results enabled true/false-positive and false-negative classifications. Because Straglr also associates each supporting read with a constituent allele in the final genotype, we could perform classification separately for the expanded and reference alleles. Classification of tandem-genotypes results was performed on all supporting reads together as it does not provide genotypes. Overall, Straglr reported a sensitivity of 99% (805/817) and perfect specificity (zero false positives), whereas tandem-genotypes showed a sensitivity of 98% (801/817) and a specificity of almost 100% (801/802). RepeatHMM identified more than the actual number of reads spanning the targeted loci in two of the three expansion sizes, suggesting the occurrence of a small number of false positives.

**Fig. 2:**
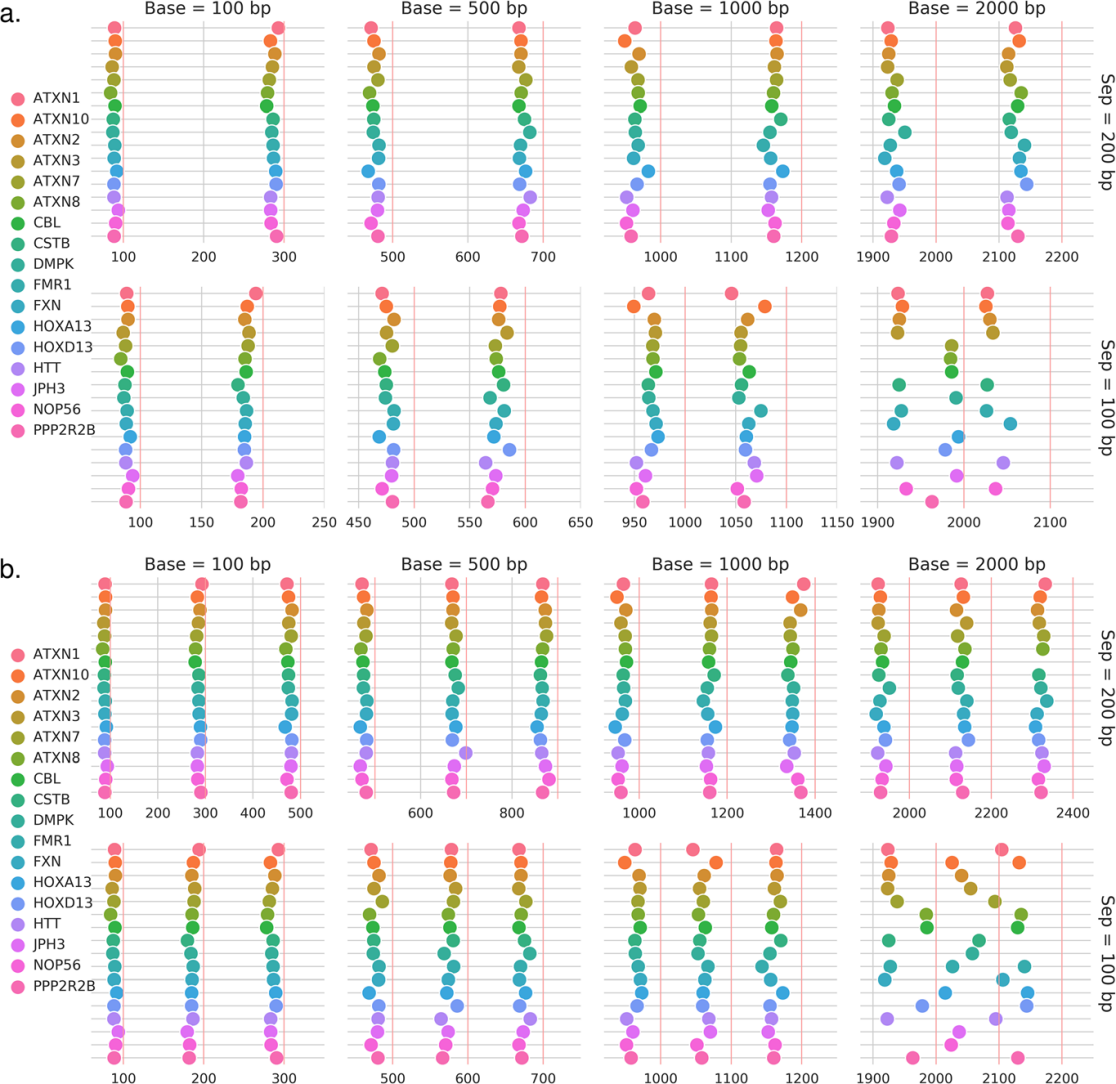
Genotyping benchmark (simulated data): resolving power. A series of bi-allelic (**a**) and tri-allelic (**b**) samples composed of a “base” expansion (columns) at 17 disease loci (legend) combined with one (**a**) or two (**b**) larger alleles separated from the next smaller allele by a fixed separation size (rows). Red vertical lines indicate the targeted allele sizes for simulation in each sample. Colored circles represent the allele sizes (*x*-axis) reported by Straglr for each locus (*y*-axis)

Next, we examined Straglr’s genotyping performance in resolving closely sized expanded STR alleles. We generated modified genomic sequences by replacing each of the 17 STR loci with “base” repeat sizes of 100, 500, 1000, or 2000 bp. For each base allele, we created separate modified genome sequences with alleles incrementally larger than the base by either 100 or 200 bp (see the “Methods” section). We generated reads from each modified genome using NanoSim, targeting an approximate 5-10X coverage for each allele. Finally, we combined the read set from each of the four base alleles with each of the two incrementally larger alleles to simulate a total of eight bi-allelic samples. Each sample was aligned with minimap2 and genotyped by Straglr and RepeatHMM, respectively; tandem-genotypes was excluded because it does not determine genotypes. Based on the genotype call at each locus, we found that Straglr could distinguish alleles separated by 200 bp at all four base sizes and alleles separated by 100 bp at three of the four smaller base sizes (100, 500, and 1,000 bp). At 2000 bp repeat size, alleles separated by 100 bp were resolved in 9/17 of the loci (Fig. 2a). RepeatHMM could not generate a genotype call for a vast number of loci in many of the experiments due to failure to detect insufficient coverage. Moreover, it could not resolve the two closely sized alleles in the majority of samples in which a genotype was actually reported (Additional file 1 - Fig. S1a). Interestingly, when RepeatHMM did report two alleles, they were often very close in size and one of them did not represent the true allele size (e.g., *ATXN1* in base size 1000 bp, separation 100 bp). We further extended the study by adding an additional allele with twice the separation size in each sample to simulate tri-allelic loci. Similar results to the bi-allelic samples were observed for Straglr (Fig. 2b). RepeatHMM did not report three alleles for any of the samples (Additional file 1 - Fig. S1b).

**Fig. 3:**
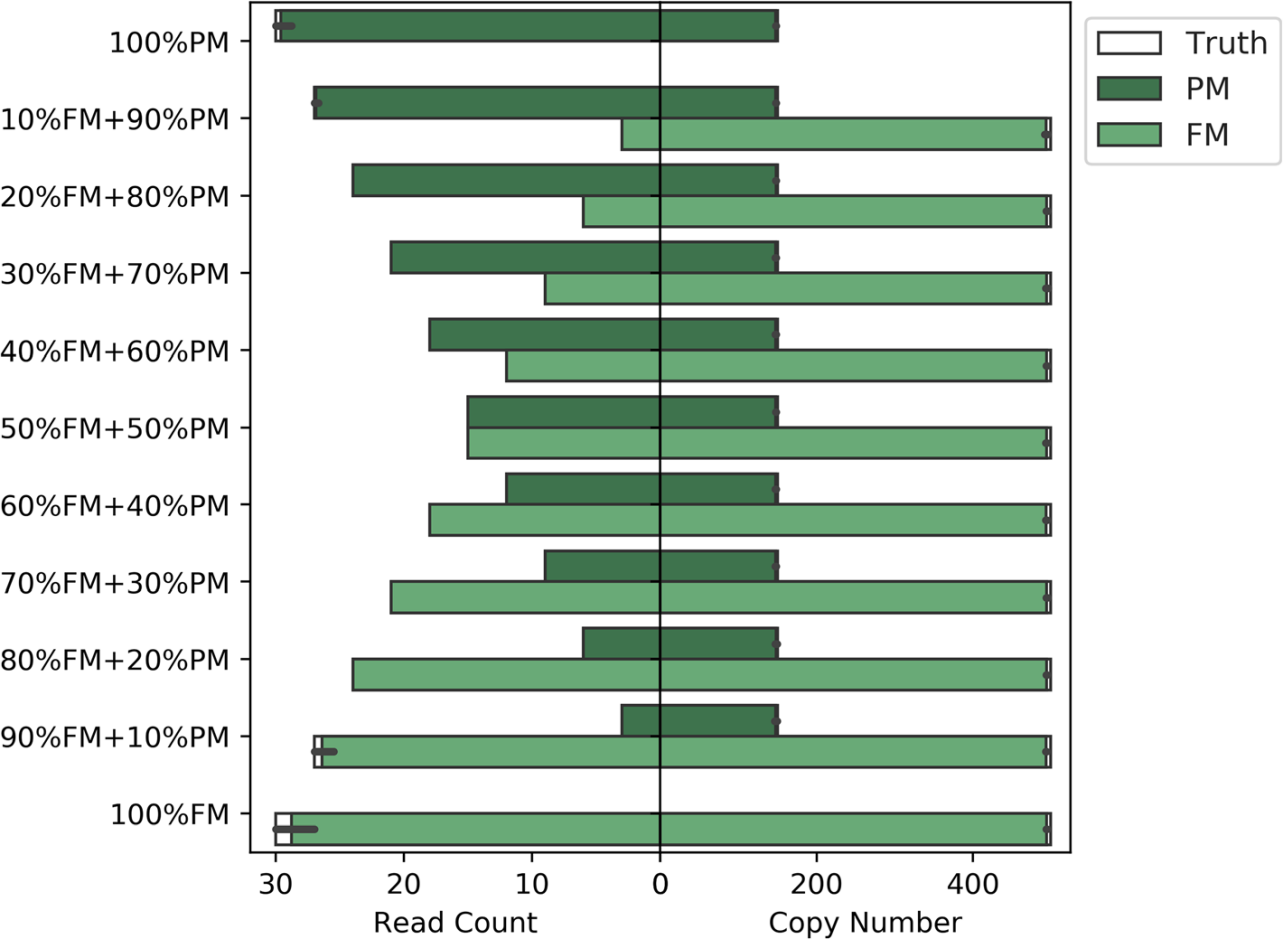
FXS mosaicism simulation. The *Y*-axis labels specify the composition of full (FM) and premutation (PM) alleles in each mosaic sample simulated. The left panel plots the number of reads simulated (white) overlaid by the number of reads assigned by Straglr for each allele (green) in each sample. The right panel plots the copy number(s) simulated (white) overlaid by Straglr’s reported copy number(s) (green) for each sample. The horizontal length of each green bar represents the average of results from ten samples of the same composition and the error bar represents the 95% confidence interval. Thirty simulated reads spanning the *FMR1* repeat locus with the specified allele composition is used as the input for each experiment. FM allele has 500 repeats and PM has 150 repeats

We also tested Straglr’s ability to detect mosaicism using simulated fragile X syndrome (FXS) samples that contained different relative abundances of a premutation (PM, 150 repeats) and full mutation (FM, 500 repeats) *FMR1* expansions. We simulated ten 30X samples for each combination of PM and FM alleles, from 100% PM progressing to 100% FM with a step-size shift of 10% read abundance from one allele to the other (Fig. 3). We showed that Straglr not only successfully detected the right allele(s) (Fig. 3, right), but also assigned each allele with the right number of supporting reads to reflect the underlying relative abundance (Fig. 3, left).

### Targeted sequencing data

**Fig. 4:**
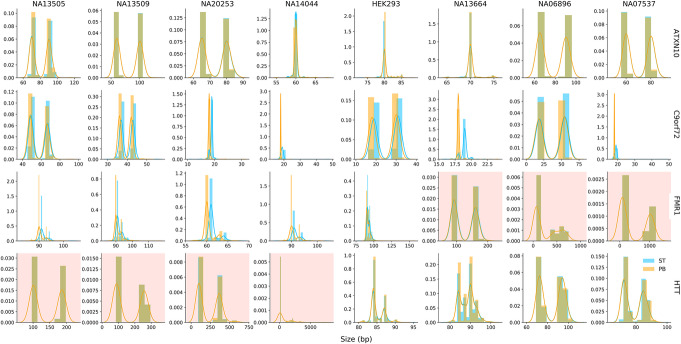
PacBio’s No-Amp targeted sequencing benchmark. Per-read repeat size distributions obtained from genotyping results of Straglr (blue) and PacBio’s repeat analysis (orange) were plotted for the eight samples (columns) in the No-Amp targeted sequencing dataset at four target loci (rows). *Y*-axis represents the density for each detected repeat size in the distribution. The four *HTT* and three *FMR1* repeat-expansion samples are highlighted by a pink background

We analyzed HiFi [3] sequence data from four samples with *HTT* CAG expansions, three samples with *FMR1* CGG expansions, and one negative control without any known repeat expansions. The data were generated by PacBio’s No-Amp targeted sequencing, an application that combines target enrichment using the CRISPR/Cas9 system with SMRT sequencing [34]. The sequences were aligned and analyzed by PacBio’s repeat analysis scripts. We ran Straglr in genotype-only mode using the provided alignments on four STR loci: *HTT*, *FMR1*, *ATXN10*, and *C9orf72*. The latter two known disease loci were included in PacBio’s analysis as negative controls. We compared the repeat size distribution on each locus extracted from Straglr’s results against those determined by PacBio’s analysis for each sample (see the “Methods” section) and observed a high level of agreement, evidenced by either complete overlaps or only minor shifts in the distributions (Fig. 4). Moreover, all expansion samples were genotyped as heterozygous, even when the separation between allele sizes was only around 100 bp. In the HTT expansion sample NA14044, Straglr also detected FM repeat sizes in excess of 1 kb.

### WGS data

For evaluation of Straglr’s performance on WGS long-read data, we used PacBio’s Sequel continuous long read (CLR) sequences (SRR7615963) and high-quality diploid assembly (GCA_003634875.1) [35] from HG00733, a sample from the 1000 Genomes Project [36]. We used the chromosome-scale, haplotype-resolved assembly as the ground-truth for examining Straglr’s performance in both targeted genotyping of TR loci and larger-than-reference TR polymorphism detection as a proxy for repeat expansions in disease scenarios.

**Fig. 5:**
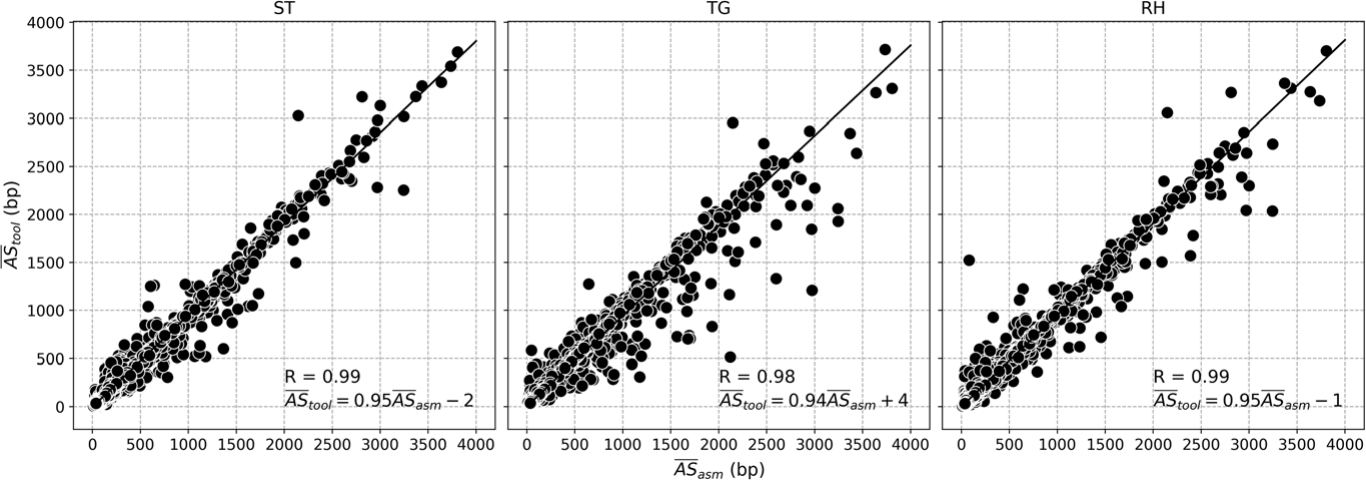
Genotyping benchmark for AQ3 HG00733: sizing accuracy. Correlation of the mean allele size (${\overline{AS}}_{tool}$) reported by Straglr (ST), tandem-genotypes (TG), and RepeatHMM (RH) against the mean allele size (${\overline{AS}}_{asm}$) determined from the HG00733 assembly between 200 and 4000 bp at 2992 annotated (hg38 Simple Repeats) loci that all three tools were able to genotype. ${\overline{AS}}_{tool}$ was calculated as the mean of all repeat sizes reported by the tool at a given locus. *R* = Pearson correlation coefficient. Linear correlation equation is shown

We first evaluated TR sizing accuracy of Straglr, tandem-genotypes, and RepeatHMM. For this, we used the UCSC hg38 “Simple Tandem Repeats” annotation [37], which is composed of both STRs and VNTRs, as the input for target loci. To streamline the analysis with minimum ambiguity, we reduced the initial set of over a million loci to ones that (A) have a motif size between 2 and 100 bp and (B) are located more than 500 bp from another TR to avoid annotated loci with different repeat motifs that overlap to various degrees. TR sizes in the assembly were determined by aligning flanking probe sequences generated from the reference genome against the assembly (see the “Methods” section). Finally, we restricted our comparison against the assembly to a set of common loci (*n* = 215,894) that all three tools were able to genotype. We plotted the mean repeat sizes determined from individual support reads against the average repeat sizes determined from each assembly scaffold for each locus. Higher Pearson correlation coefficients for Straglr and RepeatHMM (*R* = 0.99) demonstrated these tools sized TRs more accurately than tandem-genotypes (*R* = 0.98) (Fig. 5)

**Fig. 6:**
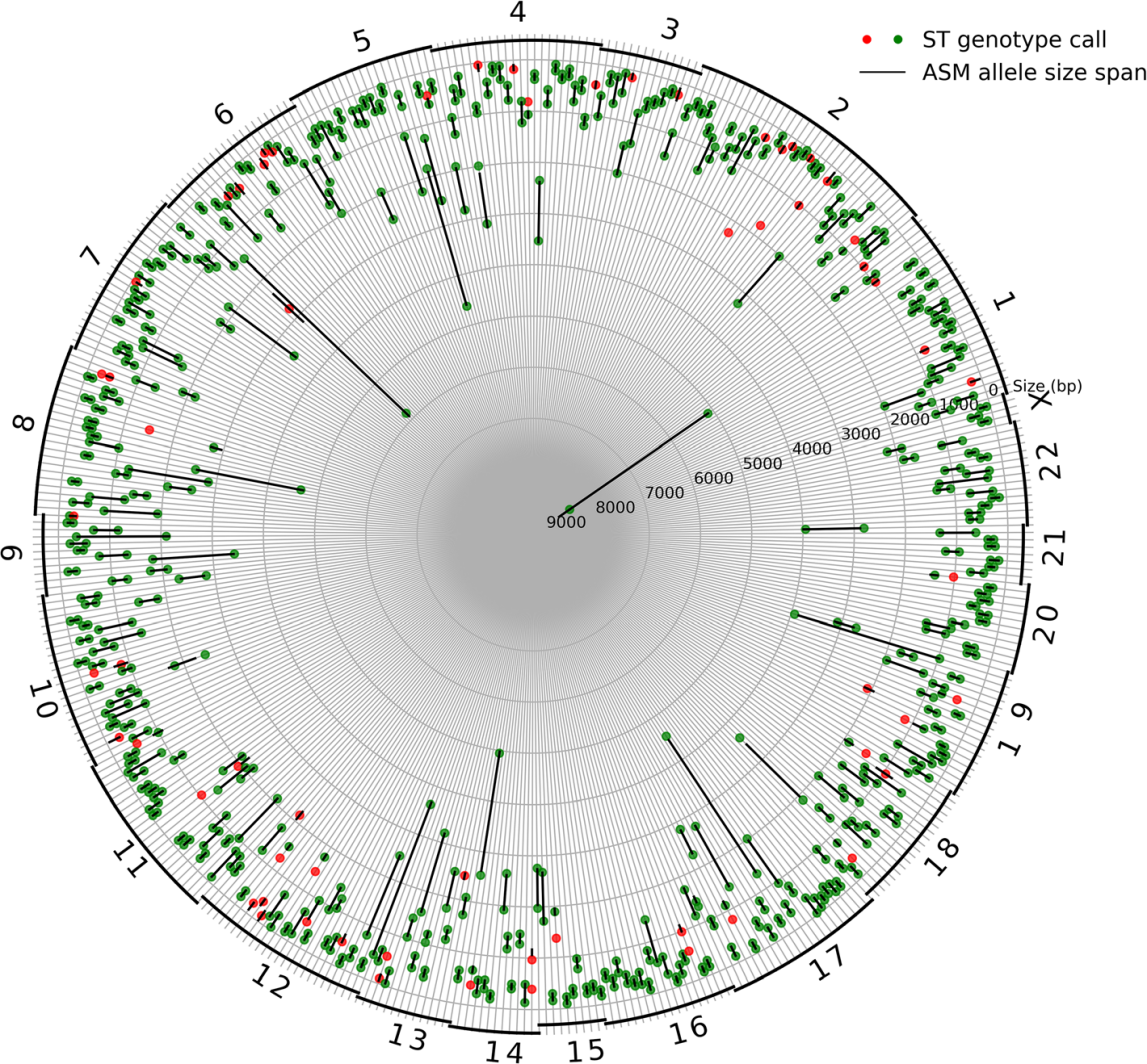
Heterozygous loci genotyping benchmark for HG00733. Comparison of allele sizes determined from the assembly against Straglr’s genotyping results for 418 annotated heterozygous loci (see the “Results” section for selection criteria). Each radial line in the circular plot represents a locus. The chromosome on which the locus lies is shown as a number and arc along the circumference. The black segment on each radial line represents the span in size (bp) between the two alleles determined from the assembly. Colored circle markers on each radial line indicate allele sizes according to Straglr’s genotype. One or two markers may be present on each radius because Straglr may only report a single allele that is found heterozygous by the assembly. Green markers represent agreement between the allele sizes (see the “Methods” section for matching criteria), red indicates disagreement

For evaluation of Straglr’s genotyping performance on WGS data, we concentrated on a subset of the annotated loci selected above that have heterozygous alleles that differ by at least 100 bp as determined from the assembly (*n* = 418). Straglr’s genotyping results (Fig. 6), also summarized in Additional file 1 – Table S1, exhibited a high level of sensitivity (87%, 364/418) and specificity (93%, 364/390) (see the “Methods” section) using the diploid assembly as the “ground truth.” This stands in sharp contrast with RepeatHMM (Additional file 1 – Table S1 and Fig. S2) with 40% (168/418) sensitivity and 84% (168/201) specificity.

With the same benchmarking framework, we also studied the relationship between Straglr’s genotyping performance and coverage depth. We generated samples with various sequencing depths by randomly sub-sampling the HG00733 dataset and genotyped the 418 heterozygous loci with Straglr. We used sensitivity, defined as the percentage of loci with both alleles genotyped within 100 bp of the assembled sizes, as the performance metric and found that genotyping performance increased linearly with coverage up to around 20X, and began to plateau after around 30X (Additional file 1 - Fig. S3).

**Fig. 7:**
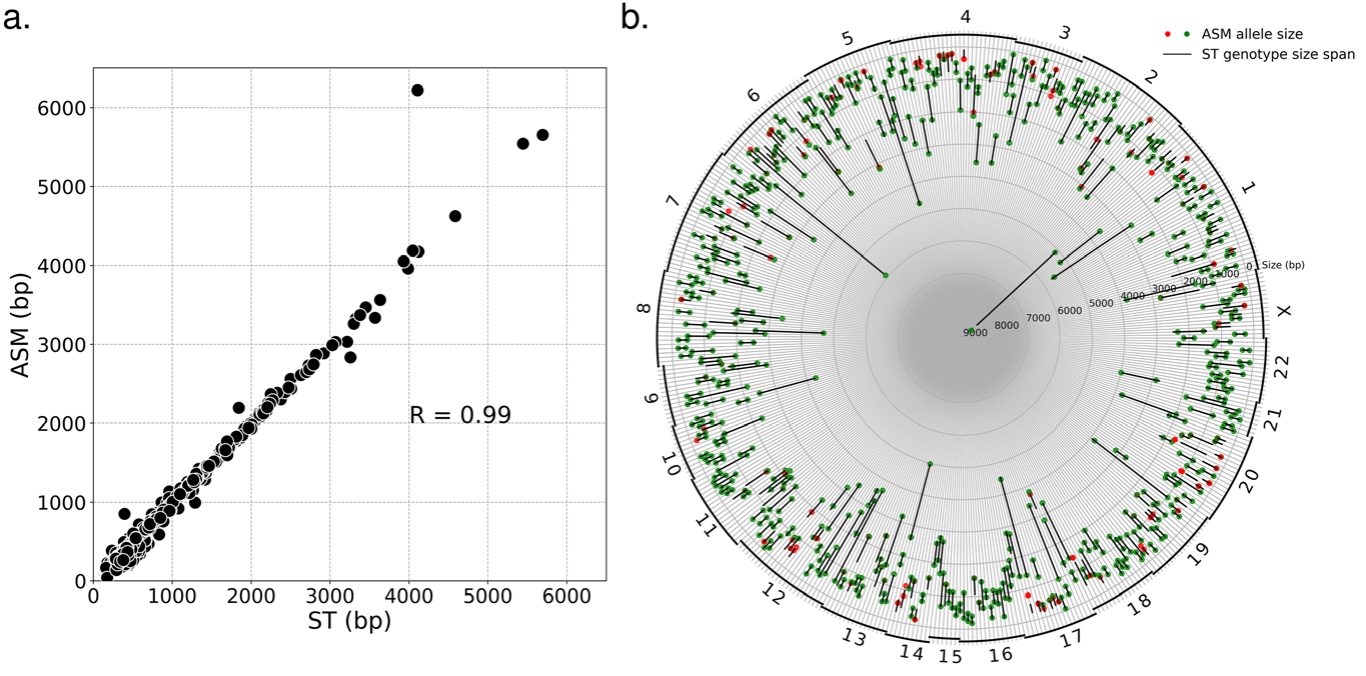
Characterization of homozygous and heterozygous expansions detected in HG00733 Straglr genome scan. **a** Correlation of TR sizes of homozygous loci (see the “Results” section for selection criteria) detected by Straglr genome scan (ST) and their corresponding sizes determined from the assembly (ASM). Averages of two alleles from the assembly were calculated and plotted because two differently sized TR may have been reconstructed in the assembly. **b** Heterozygous loci based on Straglr genome scan (see the “Results” section for selection criteria). Each radial line in the circular plot represents a locus. The chromosome on which the locus lies is shown as a number and arc along the circumference. The black segment on each radial line represents the span in size (bp) between the two alleles within Straglr’s genotype. Colored circle markers on each radial line indicate allele sizes determined from the assembly. One or two markers may be present on each radius because the assembly may only report a single allele that is found heterozygous by Straglr. Green markers represent agreement between the allele sizes (see the “Methods” section for matching criteria), red indicates disagreement

We ran Straglr in genome-scan mode on HG00733 minimap2 alignments to capture expansions of TRs with 2-100 bp motifs. To evaluate the results, we performed separate comparisons against the assembly on homozygous and heterozygous loci, classified based on Straglr’s genotyping. We selected homozygous loci with at least 20 supporting reads and larger than the reference by 100 bp (*n* = 540). We plotted the TR sizes reported by Straglr versus those determined from the assembly. The mean of the two allele sizes was used for plotting in Fig. 7 because they may be different in the assembly. A high degree of correlation (*R* = 0.99) between the allele sizes called by Straglr and the assembly was observed (Fig. 7a). From Straglr’s heterozygous calls, we selected loci with alleles that differed in size by at least 200 bp, each with minimum 20 read support, located at least 500 bp apart from each other, and for which sizes could be unambiguously determined from the assembly (*n* = 437). Of these, we observed 315 (72%) where both alleles agreed with the assembly, 106 (24%) agreed with one allele, and 16 (4%) agreed with neither (Fig. 7b). Although we used the HG00733 assembly as the “ground truth,” visual inspection of some of these 122 discordant cases raises questions about the haplotype reconstruction on some of these loci. In Additional file 1 - Fig. S4, we present an example in which one of the two alleles called by Straglr is matched while the smaller allele was missing from the assembly.

Of the 6936 loci with alleles called as “expanded” by Straglr, 146 were not annotated as simple repeats by UCSC. At one of these loci at chr9:127,882,557-127,882,603, Straglr found a stretch of nine to 10 impure GAA repeats in the reference sequence that was “expanded” to over 140 copies in HG00733. This result was confirmed by checking the corresponding locus in the assembly manually (Additional file 1 - Fig. S5). We found this example particularly interesting because it reminds us of the repeat expansion in *FXN* in Friedreich Ataxia (FRDA), which also occurs at a short (six copies) GAA tract that is not included in the UCSC simple repeat annotations.

### Runtime and computing resource

**Fig. 8:**
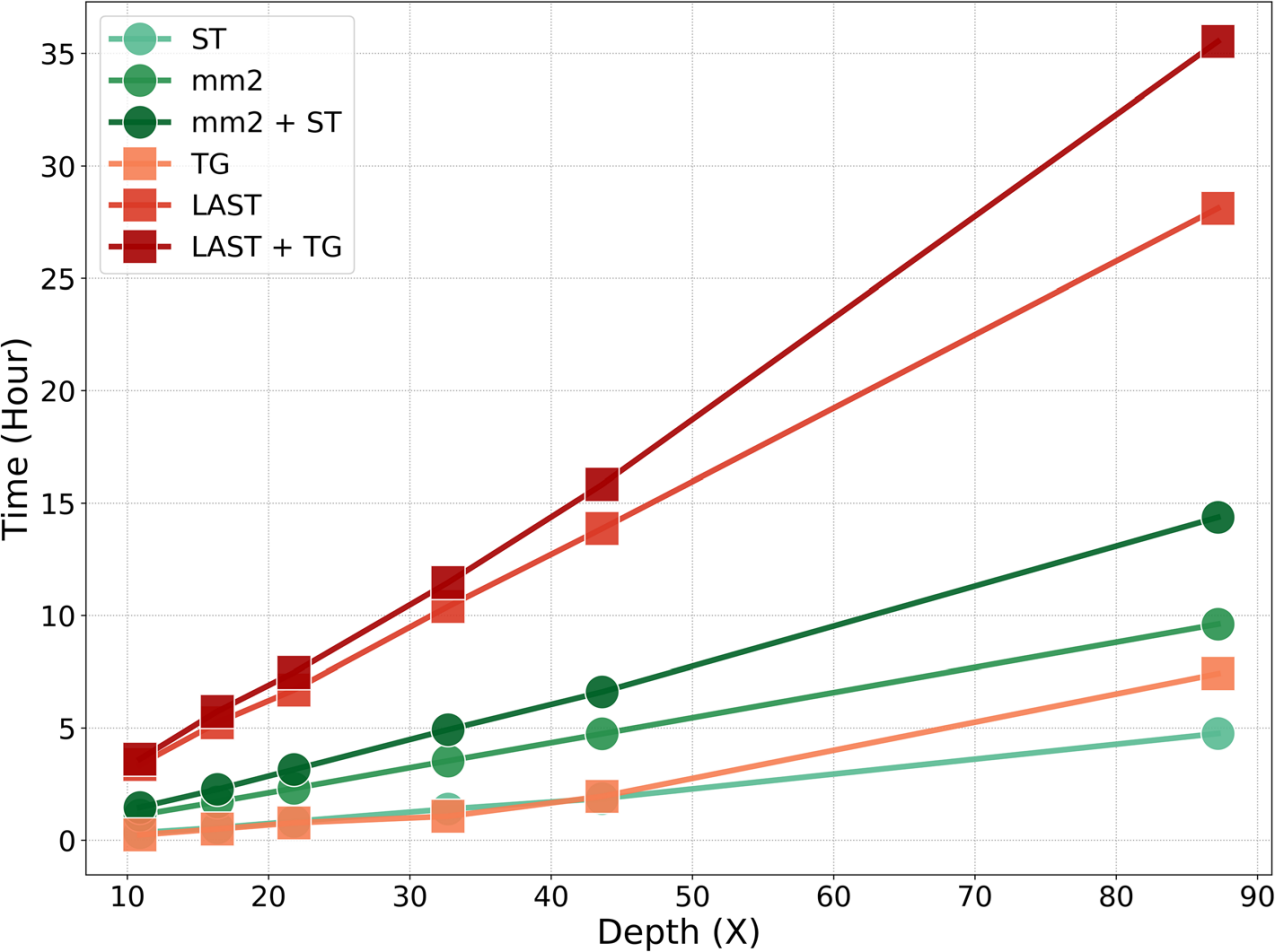
Runtime comparison. Sequences from HG00733 (accession SRR7615963) were randomly sub-sampled to generate six samples of different read depths: 11, 16, 22, 33, 44, and 87X (original library). Runtimes were shown for running the aligners alone (minimap2 or LAST), the analysis tools alone (Straglr or tandem-genotypes), and the sum (minimap2 + Straglr, LAST + tandem-genotypes) using 32 threads/processors. tandem-genotypes time is taken from the longest completion time of any one of the 32 equal-size batches (22,445 loci) generated from hg38 simple repeats. LAST alignment times are the sum of running last-train and lastal

We ran Straglr in genome-scan mode on six samples generated by randomly sub-sampling Sequel reads from HG00733 (accession SRR7615963) at different read depths: 11, 16, 22, 33, 44, and 87X (original library) to study the effect of coverage depths on runtime of the software. To provide an idea of running the entire pipeline, we also included the runtimes of minimap2 alone and the sum of the runtimes of minimap2 and Straglr in the benchmarking (Fig. 8). At around 30X depth of coverage, the target coverage in most sequencing experiments, minimap2 completed in about three hours and Straglr in about one and a half hours. For comparison, we ran LAST/tandem-genotypes pipeline on the same samples. In order to simulate a genome scan using tandem-genotypes, we divided the hg38 Simple Repeats annotation into 32 equal batches of 22,445 loci each, the same number of processors we used for Straglr, after excluding the same regions we excluded for Straglr (see the “Methods” section), and ran tandem-genotypes on them in parallel. The longest runtime for the 32 jobs was taken as the completion time of the genome scan. RepeatHMM was not included in the comparison because all batches took over 24 h to complete. For alignment alone, we found that LAST was around three times slower than minimap2 in all samples of different coverage depths. Straglr and tandem-genotypes took about the same time to run for up to 44X coverage; Straglr was a little faster for the deepest sample. At 30X, tandem-genotypes took about 1 h to finish and LAST about 10 h to finish. tandem-genotypes is more efficient in memory usage as it only took about 92 MB (peak usage) for processing the 30X sample compared to 15 GB for Straglr. This is understandable because Straglr inspects the sequence content of each target locus but tandem-genotypes does not. All computing benchmark runs were performed on Intel Xeon E5 -2650 2.20 GHz 48-core machines running Centos 7.6.

## Discussion

We designed different simulation experiments to evaluate Straglr’s genotyping performance. We first demonstrated Straglr’s superior specificity and sensitivity (see the “Results” section) in detecting a wide spectrum of TR expansions from 100 to 4000 bp for a variety of known disease loci. Then, we tested Straglr’s ability to resolve two or three closely sized alleles at various expansion sizes. We found that Straglr can readily distinguish expanded alleles (100–1000 bp in length) separated by as small as 100 bp. Smaller alleles (50 bp) separated by as small as 60 bp remain distinguishable at a majority of test loci (14/17, data not shown). At larger expansions of 2 kb or larger (data not shown), alleles different by 100 bp were still resolvable, but the success rate (based on the 17 test loci) started to decline with increasing allele sizes (data not shown). This is expected as sequencing errors, such as deletions, accumulate in larger alleles, confounding real allele size differences with sizing imprecision and hence making allele clustering to generate accurate genotypes more challenging. Nevertheless, Straglr’s ability to capture multiple heterozygous expansion alleles is important because it has been reported that algorithms using short-reads often failed to capture the second expansion allele or misclassified one of the two FM alleles as PM or normal [18]. This is undesirable for diseases with recessive inheritance, such as FRDA, because affected individuals may be wrongly diagnosed as carriers. In the final simulation experiment, we constructed samples to mimic FXS mosaicism [38] with different read dilutions of FM and PM alleles at different relative abundances for testing Straglr’s genotyping performance. We found that Straglr could accurately estimate not only the correct copy numbers of the two alleles but also their relative abundance, reflected by the number of supporting reads associated with each allele.

Straglr was developed around genome alignments produced by minimap2 [39] because of its speed and ease of use, although output from other aligners in BAM format is also accepted. Despite minimap’s overall mapping reliability, we observed the occasional omission of split alignments before or after long repeats (1 kb or more). This deficiency is of particular relevance to TR alignment, but we managed to salvage most of such cases by aligning a short stretch of reference sequence immediately upstream or downstream of the target position using BLASTN (see the “Methods” section), thereby improving Straglr’s sensitivity in capturing TRs. This also eliminated the need to re-map neighborhood sequences around TR loci using another aligner, the approach used by RepeatHMM, the slowest of the three long-read TR genotyping tools in this study.

One challenge of detecting TRs in noisy long reads is that interruptions introduced by indels and base substitutions make the task of motif recognition difficult. Running tandem repeats finder (TRF) with lenient parameters in Straglr enabled TRs with imperfect or complex motifs to be detected. Nonetheless, occasional failures may occur in short alleles where deletions can cause disruptions big enough to preclude motif recognition, an example being the normal allele of 42 bp GGCCTG in *NOP56* of spinocerebellar ataxia 36.

Another challenging task during the genotyping phase is comparison of TR motifs identified from individual locus-spanning reads against the target motif, either provided by the user or detected by Straglr during the genome-scan phase. This is particularly difficult with VNTRs as longer TR sequences are inherently more error-prone and divergent. We employed BLASTN with reduced seed length to perform matching between long TR motifs, and imperfect identity with target motifs was accepted as positive matches. This motif screening procedure added extra running time for Straglr but was necessary to avoid STR genotyping mistakes. We provide an example at chr8:23,627,962-23,628,004 in HG00733 where an insertion of ~ 3.5 kb was observed in 35 out of 86 reads (Additional file 1 - Fig. S6), but the insertion sequence does not remotely resemble a concatenation of the annotated TATC repeat and no single unique motif spanning the entire sequence could be identified by TRF. The locus is correctly reconstructed in the Falcon assembly, capturing the insertion in one of the scaffolds, but the decision to ascertain the insertion as an expansion of the TATC motif, as was done by tandem-repeats, is questionable.

Our analysis of the PacBio No-Amp targeted sequencing dataset demonstrated Straglr’s versatility in genotyping sequences generated from the HiFi sequencing technology. In fact, the almost-identical size distributions (Fig. 4) and estimates (Additional file 3) of repeats extracted from individual reads by both Straglr and PacBio’s proprietary analysis show that Straglr’s genotyping performance expectedly improves on more accurate long-read sequences. Moreover, this dataset also provides an opportunity for testing Straglr’s ability to genotype loci composed of multiple motifs, such as neighboring CAG and CGG repeats at the *HTT* locus, which are difficult to simulate for benchmarking. Straglr is flexible in allowing users to specify multiple motifs for genotyping complex loci. Together with improving accuracy in long-read sequencing technologies, Straglr can be deployed to delineate embedded interruption repeat motifs, a common phenomenon that influences phenotype manifestations in a number of repeat expansion diseases [40–42].

Simulation is a very useful benchmarking avenue as the “ground truth” is available. We believe our benchmarking results from the simulated Nanopore datasets can also be achieved on PacBio’s raw CLR sequences as both technologies exhibit similar overall error rate [4]. For this, we used the HG00733 diploid assembly to evaluate Straglr’s results on WGS long reads from the same sample. Overall, we found a high concordance (*R* = 0.99) between the sizes of annotated TRs determined by Straglr and the corresponding loci from the assembly (Fig. 5). Over 200,000 TRs with motifs ranging from 2 to 100 bp were analyzed, attesting to the ability of Straglr to genotype TRs with a wide range of purity and complexity. On a subset of annotated TRs deemed heterozygous based on the assembly, we observed a high percentage (87%) where both alleles reported by Straglr agreed in size.

We performed a genome scan on the entire SRR7615963 read set from HG00733 to search for TR expansions at least 100 bp larger than the allele in the reference genome. We conducted evaluations of identified homozygous and heterozygous “expanded” loci separately because the latter requires comparisons at both alleles. A high degree of correlation (*R* = 0.99) was observed between TR sizes from homozygous loci detected by Straglr’s genome scan and their corresponding sizes in the assembly. We observed a lower level percentage (73%) of heterozygous loci where both allele sizes agreed between Straglr and the assembly. As reported in the “Results” section, the majority of the remaining cases were single-match cases, where only one of the two alleles called by Straglr agreed with the assembly. We suspect a sizable proportion of them, an example of which shown in Fig. S3, were cases in which a second allele failed to be reconstructed in the assembly based on the number and tight size distribution of supporting reads of the unmatched allele. Other single-match cases probably contained a second false-positive allele composed of heterogeneous repeat sizes erroneously clustered together by the Gaussian mixture models (GMM) Straglr uses for genotyping. GMM generally performed well in determining TR genotypes, as evidenced by the simulation experiments and the benchmarking of HG00733’s genotypes against the long-read assembly. Preventing GMM from clustering suspiciously wide size or repeat number distributions into the final genotype might improve Straglr’s results. As long-read phasing algorithms become more mature [43, 44], allele phasing may be the most reliable solution to determining TR genotypes.

In Straglr, repeat expansions are identified in the genome-scan phase by extracting TR insertions relative to the reference genome. This allows Straglr to discover expansions at novel locations outside of pre-determined annotated TRs—input that is required by existing long-read genotyping software. Although HG00733 is a sample from a healthy individual, we found examples of apparently expanded TR alleles at unannotated loci (Additional file 1 - Fig. S5). It is doubtful that these observations have any clinical implications because only a tiny fraction of TR loci have any known association with disease and most TR loci are neither within nor adjacent to protein-coding genes [45].

Straglr is efficient at performing a genome scan, requiring only around 1 h and 23 min using 32 threads and 15 GB of memory to process genome alignments of 30X depth of coverage—an optimal coverage for genotyping we found using PacBio CLR data (see the “Results” section). With Straglr’s ability to process long reads from both PacBio and Nanopore platforms, we plan to catalog human TR polymorphisms from all publically available whole genome long-read sequencing datasets, thereby providing a control to aid in the discovery of pathogenic TR expansions in disease samples.

## Conclusions

Long-read sequencing enables reliable detection of pathogenic TR expansions, and we have demonstrated that Straglr is a robust software for such application. Adopting an approach similar to structural variant detection but customized for handling TR alignment idiosyncrasies, Straglr run in genome-scan mode efficiently identifies TR expansions relative to the reference genome. We detected larger-than-reference polymorphic TR alleles from a healthy 1000 Genomes Project sample with allele size estimates that strongly correlate with the corresponding sequence assembly. With many apparently genetic disorders still having unknown causal mutations and the technologies of long-read sequencing becoming more mature and cost-effective, we believe that Straglr is a robust tool for exploring the involvement of repeat expansion events in the etiology of genetic diseases.

## Methods

### Straglr method

**Fig. 9:**
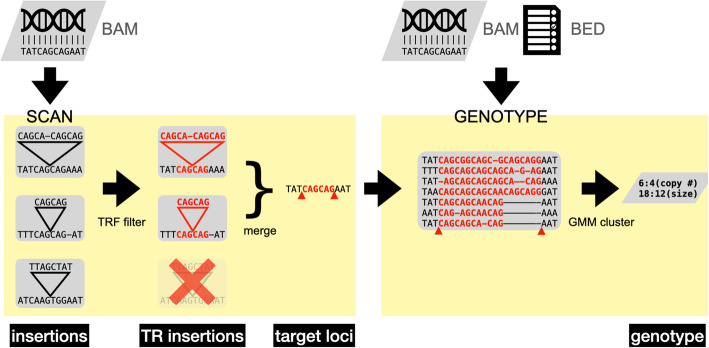
Straglr workflow. Straglr has two stages: Scan and Genotype. Inputting a bam file, the Scan stage identifies insertions, filters them for tandem repeats, and merges reads to annotate events at target loci. Inputting bam and bed files, the Genotype stage uses a Gaussian mixture model to cluster reads into alleles to report copy numbers of repeat motifs and the nucleotide lengths of tandem repeats

Straglr can be run in genome-scan or genotype-only modes (Fig. 9). The difference between these modes is that the input TR loci are identified by Straglr’s genome-wide expansion searching in the former but are provided by the user in the latter. When run in genome-scan mode, Straglr searches genomic alignments in BAM format for insertions larger than a user-specified threshold. It extracts insertions that are captured either within a single alignment, which can be readily gleaned from the CIGAR representation, or embodied in multiple split alignments. Sizable insertions or insertions positioned near the end of a read sequence often cause aligners to generate split alignments from a single read. Potential insertions in such cases can be recognized by the close proximity of the genomic positions coupled with distant separation of the read positions of the breakpoint between the paired split alignments. Straglr extracts insertions by iterating through all alignments and merges events that are within close proximity of each other. Before the start of genome-scan, Straglr accepts a list of coordinates that the user chooses to bypass. This option allows users to expedite the searching process by skipping dubious regions, such as the highly repetitive centromeres or regions harboring extensive segmental duplications, from which reliable alignments of even long reads may not be possible. Straglr then uses TRF to screen the insertion sequences of all detected events—only insertions composed of at least 70% of single TRs are kept. Straglr also extracts the neighborhood genomic region of each event and summons TRF to delineate the genomic position of the TR locus, thus eliminating the need for annotation. Motifs detected from insertion sequence and genomic region must be matched, albeit with non-stringent criteria in light of noisy read sequences, in order for events to proceed to the next stage of analysis. We enlist BLASTN to help match VNTRs because subtle discordance between read and reference genome sequences at such long loci is usually anticipated. Because of the high error rate of long reads, TRF is run with lenient parameters for TR detection (Additional file 2 - Table S2). The coordinates and sequence motifs of candidate TR expansions are recorded and passed on to the genotyping module of the software.

The genotyping module, given a list of coordinates and motifs either from the genome-scan module or input by the user in BED format to run Straglr in genotype-only mode, iterates through each locus and extracts read sequences sandwiched between the coordinates of the neighboring nucleotides. A similar strategy is used to extract insertion sequence from single or split alignments obtained from Straglr’s genome-scan module. Straglr attempts to rescue missing split alignments in which there are potential TR sub-sequences by aligning a short stretch (80 bp) of reference genomic sequence immediately up- or downstream of the position where the possible sub-sequence lies to the clipped read sequence using BLASTN. A successful unambiguous alignment provides the missing boundary of the repeat sub-sequence with the read and enables Straglr to extract the sequence for TRF inspection. TRF is again used to screen all candidate repeat sequences extracted, as motifs detected are matched against the target motif. When simple string matching using Python’s regular expression fails, such as the cases for long and complicated VNTRs, BLASTN is performed with mismatch allowance to discern matches between target and detected motifs. If none of the detected motifs matches the target motif, no genotyping result will be produced for the locus in question. The final size and motif sequence for each supporting read are extrapolated from TRF results. This process captures potential TRs smaller than the alleles identified from genome-scan mode (if genome-scan mode was run), hence compiling a complete list of alleles for genotype ascertainment.

Straglr uses GMM (Python scikit-learn package) to estimate the genotype given all the repeat sizes identified at each target locus. It attempts different numbers of clusters up to a user-specified maximum (default = 2) and assigns the one with the smallest Akaike information criterion (AIC) value as the number of alleles. The median of all repeat sizes within each cluster is the size reported for that allele. The final output of Straglr details the supporting read names together with the copy numbers, sizes, and start location of the TR detected in each read.

### Tandem repeat expansion simulation

To simulate TR expansions, we replaced specific TRs (Table 1) in the reference genome with sequences of desired lengths made up of additional copies of the original repeat motifs. Simulated Nanopore sequencing reads were generated using NanoSim (v2.5.0) with the Guppy flip-flop model it provides. For the first experiment that tested Straglr’s accuracy in capturing expansion events, we performed whole genome simulations and generated 3,700,000 reads; for the second experiment that tested Straglr’s resolving power for expansion alleles, we only performed locus-specific simulation on the expanded loci with 500 kb flanking sequences and a target number of 10,000 reads. This was done to expedite the data generation process in light of the multitude of simulations designed. Each sample in the first experiment was composed of reads generated from a modified genome and reads generated from the reference genome. Samples in the second experiment were made up reads generated from two or three modified genomes with different expansion sizes. All samples were targeted for 20-30X coverage. Each sample was aligned with minimap2 and analyzed by Straglr in genotyping mode provided with locus coordinates and motifs, as well as by tandem-genotypes and RepeatHMM where appropriate.

To identify reads that spanned the targeted loci modified with expansions, the origin of each simulated read was first deduced from NanoSim’s read names: in the underscore-separated fields of the read name, the first field represents the chromosome name, the second field represents the start genomic coordinate, the third field indicates whether the read is mappable to the genome (“aligned”) or not (“unaligned”), and the seventh field represents the alignment length of the read, which by adding to the start coordinate yields the end genomic coordinate of the read. The coordinates of all “aligned” reads were intersected with the target locus coordinates with expansion sizes added to the ends, and the subsets that overlapped by at least 50 bp on both sides of the expansions were treated as the set of ground truth reads that harbor the expansions.

### Mosaicism simulation

We generated two modified genome sequences, one replacing the *FMR1* repeat locus (chrX: 147,912,051–147,912,110) with 150 CGG repeats (PM) and the other with 500 repeats (FM), each with 500 kb reference sequences flanking the locus. We then generated 2000 simulated Nanopore reads by running NanoSim on each modified sequence, aiming to result in approximately 30 reads covering the locus. This step was repeated ten times to generate inputs for each mosaic composition. We then aligned all simulated reads against hg38 with minimap2 and identified all PM and FM reads that flanked the *FMR1* repeat locus by at least one kb on both sides. From these sets, reads were randomly picked to make up the desired composition for each sample with a target combined coverage of 30X. Starting with samples composed of 100% PM reads, we produced a series of samples by serially shifting 10% of the input reads from PM to FM until the final samples that were composed of 100% FM reads. Each sample was aligned with minimap2 and genotyped by Straglr. The genotype output columns, with both the copy numbers (numbers of repeat units) detected and the number of supporting reads associated with each allele, were used as the inputs for generating Fig. 3.

### PacBio’s No-Amp targeted sequencing analysis

Alignments and analysis results were downloaded from PacBio’s No-Amp Targeted Sequencing dataset repository: https://downloads.pacbcloud.com/public/dataset/RepeatExpansionDisorders_NoAmp/. In the “analysis” sub-directory, BAM files used for Straglr analysis resided under “align,” and per-read repeat sizes were extracted from the “totalLength” columns of the “*.counts.tsv” files under “reports.” Per-read repeat sizes determined by Straglr were taken from the “size” field of its outputs. Target repeat coordinates and motifs used for running Straglr in genotype-only mode were copied from the “human_hs37d5.targets_repeatonly.bed” file under the “auxillary” sub-directory.

### Tandem repeat size determination from assembly

To determine TR sizes from the assembly, we extracted 500 bp reference genome sequences flanking either side of the TR coordinates (left and right probes) and aligned them against the assembly with BWA mem [46]. Only unique, end-to-end mappings were considered for determining the positions of TRs within the assembly. The end mapping position of the left probe and the start mapping position of the right probe were taken as the boundaries of a given TR within the scaffold both probes mapped to. The assembly sequences bounded by the coordinates thus determined were subject to testing by TRF to ensure at least 80% of the extracted sequence was composed of a single TR. Assembly sizes were only reported for TRs that passed this test.

### Comparison between Straglr’s genotype and the assembly

To determine if heterozygous alleles of a given TR locus ascertained by either Straglr or RepeatHMM agreed with the corresponding alleles from the diploid HG00733 assembly, we sorted the allele sizes and compared the smaller and larger alleles from each source independently. A match was declared when both alleles were within 20 bp in size or the difference was within 10% of the assembly allele size. True positives were represented by heterozygous genotype calls in which both alleles matched the assembly using the above criteria, with the total number of heterozygous calls or the total number of heterozygous alleles identified in the assembly as the denominator in the calculation for sensitivity and specificity respectively.

## Supplementary Information

**Additional file 1.** Supplementary Figs. S1-S6 and Table S1. Supplementary benchmarking results.

**Additional file 2.** Supplementary Table S2. Software versions and parameters used in all analysis in this manuscript.

**Additional file 3.** Size estimates of target loci from both PacBio proprietary analysis and Straglr for all sequencing reads in No-Amp targeted sequencing data. (TSV 862 kb)

**Additional file 4.** Review history.

## Data Availability

Straglr is available at https://github.com/bcgsc/straglr [47] and archived under DOI 10.5281/zenodo.5090372 [48]. It is released under the GNU General Public License version 3 (GPLv3). Targeted sequencing data were downloaded from PacBio’s data repository: https://downloads.pacbcloud.com/public/dataset/RepeatExpansionDisorders_NoAmp/. The HG00733 assembly was downloaded from https://downloads.pacbcloud.com/public/dataset/HG00733_assembly/HG00733_diploid_phased_scaffolds.fasta, and the read sequences were downloaded from the Sequence Read Archive (SRA) under the accession SRR7615963. Simulation data is available upon request.

## References

[1] Mantere T, Kersten S, Hoischen A (2019). Long-read sequencing emerging in medical genetics. Front Genet.

[2] Shafin K, Pesout T, Lorig-Roach R, Haukness M, Olsen HE, Bosworth C, Armstrong J, Tigyi K, Maurer N, Koren S, Sedlazeck FJ, Marschall T, Mayes S, Costa V, Zook JM, Liu KJ, Kilburn D, Sorensen M, Munson KM, Vollger MR, Monlong J, Garrison E, Eichler EE, Salama S, Haussler D, Green RE, Akeson M, Phillippy A, Miga KH, Carnevali P, Jain M, Paten B (2020). Nanopore sequencing and the Shasta toolkit enable efficient de novo assembly of eleven human genomes. Nat Biotechnol.

[3] Wenger AM, Peluso P, Rowell WJ, Chang PC, Hall RJ, Concepcion GT, Ebler J, Fungtammasan A, Kolesnikov A, Olson ND, Töpfer A, Alonge M, Mahmoud M, Qian Y, Chin CS, Phillippy AM, Schatz MC, Myers G, DePristo MA, Ruan J, Marschall T, Sedlazeck FJ, Zook JM, Li H, Koren S, Carroll A, Rank DR, Hunkapiller MW (2019). Accurate circular consensus long-read sequencing improves variant detection and assembly of a human genome. Nat Biotechnol.

[4] Dohm JC, Peters P, Stralis-Pavese N, Himmelbauer H (2020). Benchmarking of long-read correction methods. NAR Genomics and Bioinformatics.

[5] Logsdon GA, Vollger MR, Eichler EE (2020). Long-read human genome sequencing and its applications. Nat Rev Genet.

[6] Ryan CP (2019). Tandem repeat disorders. Evol Med Public Health.

[7] Harper PS, Harley HG, Reardon W, Shaw DJ (1992). Anticipation in myotonic dystrophy: new light on an old problem. Am J Hum Genet.

[8] Paulson H (2018). Repeat expansion diseases. Handb Clin Neurol.

[9] De Roeck A, Duchateau L, Van Dongen J, Cacace R, Bjerke M, Van den Bossche T, Cras P, Vandenberghe R, De Deyn PP, Engelborghs S (2018). An intronic VNTR affects splicing of ABCA7 and increases risk of Alzheimer’s disease. Acta Neuropathol.

[10] Katsumata Y, Fardo DW, Bachstetter AD, Artiushin SC, Wang WX, Wei A, Brzezinski LJ, Nelson BG, Huang Q, Abner EL, Anderson S, Patel I, Shaw BC, Price DA, Niedowicz DM, Wilcock DW, Jicha GA, Neltner JH, van Eldik LJ, Estus S, Nelson PT (2020). Alzheimer disease pathology-associated polymorphism in a complex variable number of tandem repeat region within the MUC6 gene, near the AP2A2 gene. J Neuropathol Exp Neurol.

[11] Lalioti MD, Scott HS, Antonarakis SE (1999). Altered spacing of promoter elements due to the dodecamer repeat expansion contributes to reduced expression of the cystatin B gene in EPM1. Hum Mol Genet.

[12] Kobayashi H, Abe K, Matsuura T, Ikeda Y, Hitomi T, Akechi Y, Habu T, Liu W, Okuda H, Koizumi A (2011). Expansion of intronic GGCCTG hexanucleotide repeat in NOP56 causes SCA36, a type of spinocerebellar ataxia accompanied by motor neuron involvement. Am J Hum Genet.

[13] Rajan-Babu IS, Lian M, Cheah FSH, Chen M, Tan ASC, Prasath EB, Loh SF, Chong SS (2017). FMR1 CGG repeat expansion mutation detection and linked haplotype analysis for reliable and accurate preimplantation genetic diagnosis of fragile X syndrome. Expert Rev Mol Med.

[14] Treangen TJ, Salzberg SL (2011). Repetitive DNA and next-generation sequencing: computational challenges and solutions. Nat Rev Genet.

[15] Dashnow H, Lek M, Phipson B, Halman A, Sadedin S, Lonsdale A, Davis M, Lamont P, Clayton JS, Laing NG, MacArthur DG, Oshlack A (2018). STRetch: detecting and discovering pathogenic short tandem repeat expansions. Genome Biol.

[16] Tankard RM, Bennett MF, Degorski P, Delatycki MB, Lockhart PJ, Bahlo M (2018). Detecting expansions of tandem repeats in cohorts sequenced with short-read sequencing data. Am J Hum Genet.

[17] Dolzhenko E, Deshpande V, Schlesinger F, Krusche P, Petrovski R, Chen S, Emig-Agius D, Gross A, Narzisi G, Bowman B, Scheffler K, van Vugt JJFA, French C, Sanchis-Juan A, Ibáñez K, Tucci A, Lajoie BR, Veldink JH, Raymond FL, Taft RJ, Bentley DR, Eberle MA (2019). ExpansionHunter: a sequence-graph based tool to analyze variation in short tandem repeat regions. Bioinformatics.

[18] Rajan-Babu IS, Peng JJ, Chiu R, IMAGINE Study, CAUSES Study, Li C, et al. Genome-wide sequencing as a first-tier screening test for short tandem repeat expansions. Genome Med. 2021. 10.1186/s13073-021-00932-9. 10.1186/s13073-021-00932-9 PMC8351082 34372915

[19] Mitsuhashi S, Matsumoto N (2020). Long-read sequencing for rare human genetic diseases. J Hum Genet.

[20] Browne PD, Nielsen TK, Kot W, Aggerholm A, Gilbert MTP, Puetz L, et al. GC bias affects genomic and metagenomic reconstructions, underrepresenting GC-poor organisms. Gigascience. 2020;9(2). 10.1093/gigascience/giaa008. 10.1093/gigascience/giaa008 PMC7016772 32052832

[21] Teng JLL, Yeung ML, Chan E, Jia L, Lin CH, Huang Y, Tse H, Wong SSY, Sham PC, Lau SKP, Woo PCY (2017). PacBio but not Illumina technology can achieve fast, accurate and complete closure of the high GC, complex Burkholderia pseudomallei two-chromosome genome. Front Microbiol.

[22] Liu Q, Zhang P, Wang D, Gu W, Wang K (2017). Interrogating the “unsequenceable” genomic trinucleotide repeat disorders by long-read sequencing. Genome Med.

[23] Mitsuhashi S, Frith MC, Mizuguchi T, Miyatake S, Toyota T, Adachi H, Oma Y, Kino Y, Mitsuhashi H, Matsumoto N (2019). Tandem-genotypes: robust detection of tandem repeat expansions from long DNA reads. Genome Biol.

[24] Hoijer I, Tsai YC, Clark TA, Kotturi P, Dahl N, Stattin EL, Bondeson ML, Feuk L, Gyllensten U, Ameur A (2018). Detailed analysis of HTT repeat elements in human blood using targeted amplification-free long-read sequencing. Hum Mutat.

[25] Höijer I, Johansson J, Gudmundsson S, Chin C-S, Bunikis I, Häggqvist S, Emmanouilidou A, Wilbe M, den Hoed M, Bondeson M-L: Amplification-free long read sequencing reveals unforeseen CRISPR-Cas9 off-target activity**.** *bioRxiv* 2020. 10.1186/s13059-020-02206-w PMC7706270 33261648

[26] Nakamura H, Doi H, Mitsuhashi S, Miyatake S, Katoh K, Frith MC, Asano T, Kudo Y, Ikeda T, Kubota S, Kunii M, Kitazawa Y, Tada M, Okamoto M, Joki H, Takeuchi H, Matsumoto N, Tanaka F (2020). Long-read sequencing identifies the pathogenic nucleotide repeat expansion in RFC1 in a Japanese case of CANVAS. J Hum Genet.

[27] Sone J, Mitsuhashi S, Fujita A, Mizuguchi T, Hamanaka K, Mori K, Koike H, Hashiguchi A, Takashima H, Sugiyama H, Kohno Y, Takiyama Y, Maeda K, Doi H, Koyano S, Takeuchi H, Kawamoto M, Kohara N, Ando T, Ieda T, Kita Y, Kokubun N, Tsuboi Y, Katoh K, Kino Y, Katsuno M, Iwasaki Y, Yoshida M, Tanaka F, Suzuki IK, Frith MC, Matsumoto N, Sobue G (2019). Long-read sequencing identifies GGC repeat expansions in NOTCH2NLC associated with neuronal intranuclear inclusion disease. Nat Genet.

[28] van Kuilenburg ABP, Tarailo-Graovac M, Richmond PA, Drogemoller BI, Pouladi MA, Leen R, Brand-Arzamendi K, Dobritzsch D, Dolzhenko E, Eberle MA (2019). Glutaminase deficiency caused by short tandem repeat expansion in GLS. N Engl J Med.

[29] Trost B, Engchuan W, Nguyen CM, Thiruvahindrapuram B, Dolzhenko E, Backstrom I, Mirceta M, Mojarad BA, Yin Y, Dov A, Chandrakumar I, Prasolava T, Shum N, Hamdan O, Pellecchia G, Howe JL, Whitney J, Klee EW, Baheti S, Amaral DG, Anagnostou E, Elsabbagh M, Fernandez BA, Hoang N, Lewis MES, Liu X, Sjaarda C, Smith IM, Szatmari P, Zwaigenbaum L, Glazer D, Hartley D, Stewart AK, Eberle MA, Sato N, Pearson CE, Scherer SW, Yuen RKC (2020). Genome-wide detection of tandem DNA repeats that are expanded in autism. Nature.

[30] Corbett MA, Kroes T, Veneziano L, Bennett MF, Florian R, Schneider AL, Coppola A, Licchetta L, Franceschetti S, Suppa A, Wenger A, Mei D, Pendziwiat M, Kaya S, Delledonne M, Straussberg R, Xumerle L, Regan B, Crompton D, van Rootselaar AF, Correll A, Catford R, Bisulli F, Chakraborty S, Baldassari S, Tinuper P, Barton K, Carswell S, Smith M, Berardelli A, Carroll R, Gardner A, Friend KL, Blatt I, Iacomino M, di Bonaventura C, Striano S, Buratti J, Keren B, Nava C, Forlani S, Rudolf G, Hirsch E, Leguern E, Labauge P, Balestrini S, Sander JW, Afawi Z, Helbig I, Ishiura H, Tsuji S, Sisodiya SM, Casari G, Sadleir LG, van Coller R, Tijssen MAJ, Klein KM, van den Maagdenberg AMJM, Zara F, Guerrini R, Berkovic SF, Pippucci T, Canafoglia L, Bahlo M, Striano P, Scheffer IE, Brancati F, Depienne C, Gecz J (2019). Intronic ATTTC repeat expansions in STARD7 in familial adult myoclonic epilepsy linked to chromosome 2. Nat Commun.

[31] Yeetong P, Pongpanich M, Srichomthong C, Assawapitaksakul A, Shotelersuk V, Tantirukdham N, Chunharas C, Suphapeetiporn K, Shotelersuk V TTTCA repeat insertions in an intron of YEATS2 in benign adult familial myoclonic epilepsy type 4. Brain 2019, 142**:**3360-3366, 11, DOI: 10.1093/brain/awz267. 10.1093/brain/awz267 31539032

[32] Yang C, Chu J, Warren RL, Birol I (2017). NanoSim: nanopore sequence read simulator based on statistical characterization. Gigascience.

[33] Wick RR, Judd LM, Holt KE (2019). Performance of neural network basecalling tools for Oxford Nanopore sequencing. Genome Biol.

[34] Tsai Y-C, Greenberg D, Powell J, Höijer I, Ameur A, Strahl M, Ellis E, Jonasson I, Pinto RM, Wheeler VC: Amplification-free, CRISPR-Cas9 targeted enrichment and SMRT sequencing of repeat-expansion disease causative genomic regions**.** *bioRxiv* 2017**:**203919.

[35] Kronenberg ZN, Hall RJ, Hiendleder S, Smith TP, Sullivan ST, Williams JL, et al. FALCON-phase: integrating PacBio and Hi-C data for phased diploid genomes. *BioRxiv*. 2018;327064.

[36] Auton A, Brooks LD, Durbin RM, Garrison EP, Kang HM, Korbel JO, Marchini JL, McCarthy S, GA MV, Abecasis GR, Genomes Project C (2015). A global reference for human genetic variation. Nature.

[37] Benson G (1999). Tandem repeats finder: a program to analyze DNA sequences. Nucleic Acids Res.

[38] Dobkin CS, Nolin SL, Cohen I, Sudhalter V, Bialer MG, Ding XH, Jenkins EC, Zhong N, Brown WT (1996). Tissue differences in fragile X mosaics: mosaicism in blood cells may differ greatly from skin. Am J Med Genet.

[39] Li H (2018). Minimap2: pairwise alignment for nucleotide sequences. Bioinformatics.

[40] McFarland KN, Liu J, Landrian I, Gao R, Sarkar PS, Raskin S, Moscovich M, Gatto EM, Teive HA, Ochoa A (2013). Paradoxical effects of repeat interruptions on spinocerebellar ataxia type 10 expansions and repeat instability. Eur J Hum Genet.

[41] Pesovic J, Peric S, Brkusanin M, Brajuskovic G, Rakocevic-Stojanovic V, Savic-Pavicevic D (2018). Repeat interruptions modify age at onset in myotonic dystrophy type 1 by stabilizing DMPK expansions in somatic cells. Front Genet.

[42] Villate O, Ibarluzea N, Maortua H, de la Hoz AB, Rodriguez-Revenga L, Izquierdo-Alvarez S, Tejada MI (2020). Effect of AGG interruptions on FMR1 maternal transmissions. Front Mol Biosci.

[43] Ebler J, Haukness M, Pesout T, Marschall T, Paten B (2019). Haplotype-aware diplotyping from noisy long reads. Genome Biol.

[44] Schrinner SD, Mari RS, Ebler J, Rautiainen M, Seillier L, Reimer JJ, Usadel B, Marschall T, Klau GW (2020). Haplotype threading: accurate polyploid phasing from long reads. Genome Biol.

[45] Duitama J, Zablotskaya A, Gemayel R, Jansen A, Belet S, Vermeesch JR, Verstrepen KJ, Froyen G (2014). Large-scale analysis of tandem repeat variability in the human genome. Nucleic Acids Res.

[46] Li H: Aligning sequence reads, clone sequences and assembly contigs with BWA-MEM**.** *arXiv preprint arXiv:13033997* 2013.

[47] Chiu R, IS Rajan-Babu, Friedman JM, Birol I. Straglr: Short-tandem repeat genotyping using long reads. GitHub. 2021. https://github.com/bcgsc/straglr. 10.1186/s13059-021-02447-3 PMC8361843 34389037

[48] Chiu R, Rajan-Babu IS, Friedman JM, Birol I. Straglr: Short-tandem repeat genotyping using long reads. 2021. 10.5281/zenodo.5090372.

