# Supplementary material for: Straglr: discovering and genotyping tandem repeat expansions using whole genome long-read sequences
Source: Genome Biol. 2021 Aug 13;22:224. doi: 10.1186/s13059-021-02447-3 (PMC8361843; doi:10.1186/s13059-021-02447-3)
Supplement: Supplementary file 1 — Additional file 1. Supplementary Figs. S1-S6 and Table S1. Supplementary benchmarking results. [file 13059_2021_2447_MOESM1_ESM.docx]

a.

b.

**Figure S1. Genotyping benchmark (simulated data): resolving power of RepeatHMM.** A series of bi-allelic (**a**) and tri-allelic (**b**) samples composed of a “base” expansion (columns) at 16 disease loci (legend) combined with one (**a**) or two (**b**) larger alleles separated from the next smaller allele by a fixed separation size (rows). Red vertical lines indicate the targeted allele sizes for simulation in each sample. Colored circles represent the allele sizes (x-axis) reported by RepeatHMM for each locus (y-axis). Genotypes cannot be estimated for *CSTB* (detectable by Straglr) for all samples and therefore excluded from the plots.

|  | Straglr | RepeatHMM |
| --- | --- | --- |
| Total loci called | 418 (100%) | 290 (69%) |
| Heterozygous calls | 390 (93%) | 201 (69%) |
| double matches | 364 (93%) | 168 (84%) |
| single match | 18 (5%) | 24 (12%) |
| no match | 2 (2%) | 9 (4%) |
| Homozygous calls | 28 (7%) | 89 (31%) |
| “intermediate” | 27 (96%) | 53 (60%) |

**Table S1. Straglr genotyping results of HG00733 heterozygous loci by Straglr and RepeatHMM.** Allele sizes at 418 heterozygous loci in the diploid assembly GCA_003634875.1 were compared against Straglr’s genotyping results at the same loci. Matching criteria for allele sizes are described in ***Comparison between Straglr’s genotype*** ***and the assembly*** of **Methods**. “intermediate” indicates the single size reported by Straglr is in between the sizes of the two alleles assembled. For Straglr, single instead of double alleles reported at heterozygous loci happened usually because of flaws in the clustering stage due to insufficient supporting reads for one of the alleles rather than mistakes in repeat size estimation.

**
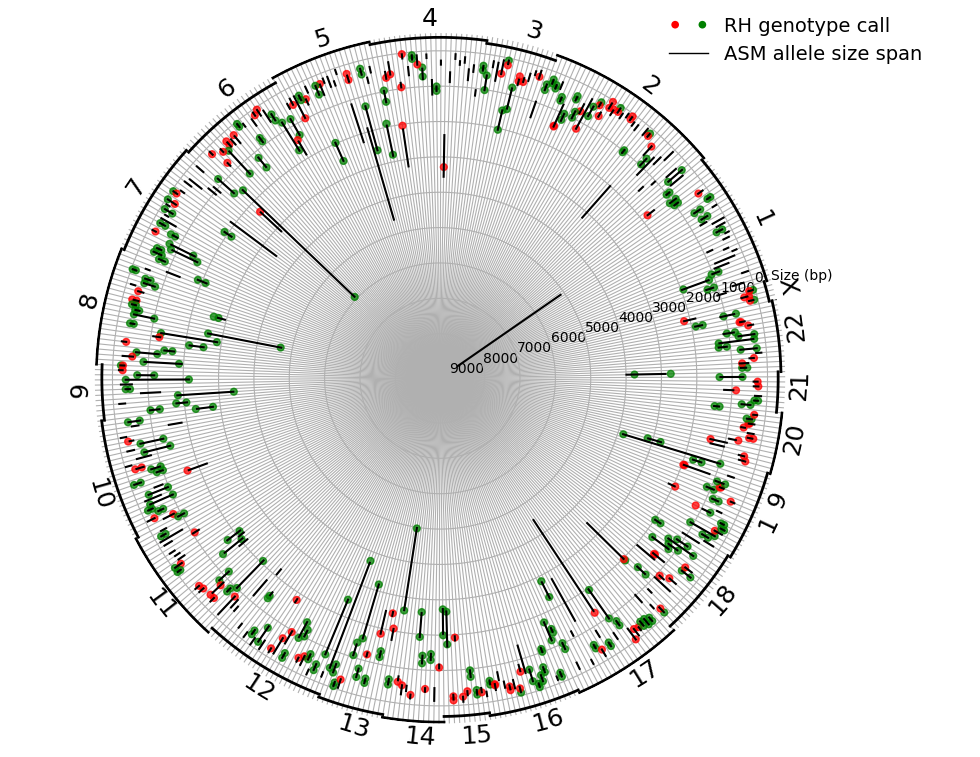
**

**Figure S2. RepeatHMM genotypes of selected HG00733 heterozygous loci.** Comparison between heterozygous alleles determined from the HG00733 assembly and from RepeatHMM genotype calls at heterozygous loci as determined from the assembly (see **Results** for selection criteria). Each radial line in the circular plot represents a locus. Black segment on each radial line represents the span in size between the two alleles determined from the assembly. Colored circle markers on each radial line indicate TR sizes extracted from RepeatHMM genotype. One or two markers may be present on each radius because RepeatHMM may only report a single allele that is found heterozygous by the assembly. Green markers represent agreement between the allele sizes (see **Methods** for matching criteria), red indicates disagreement.

**
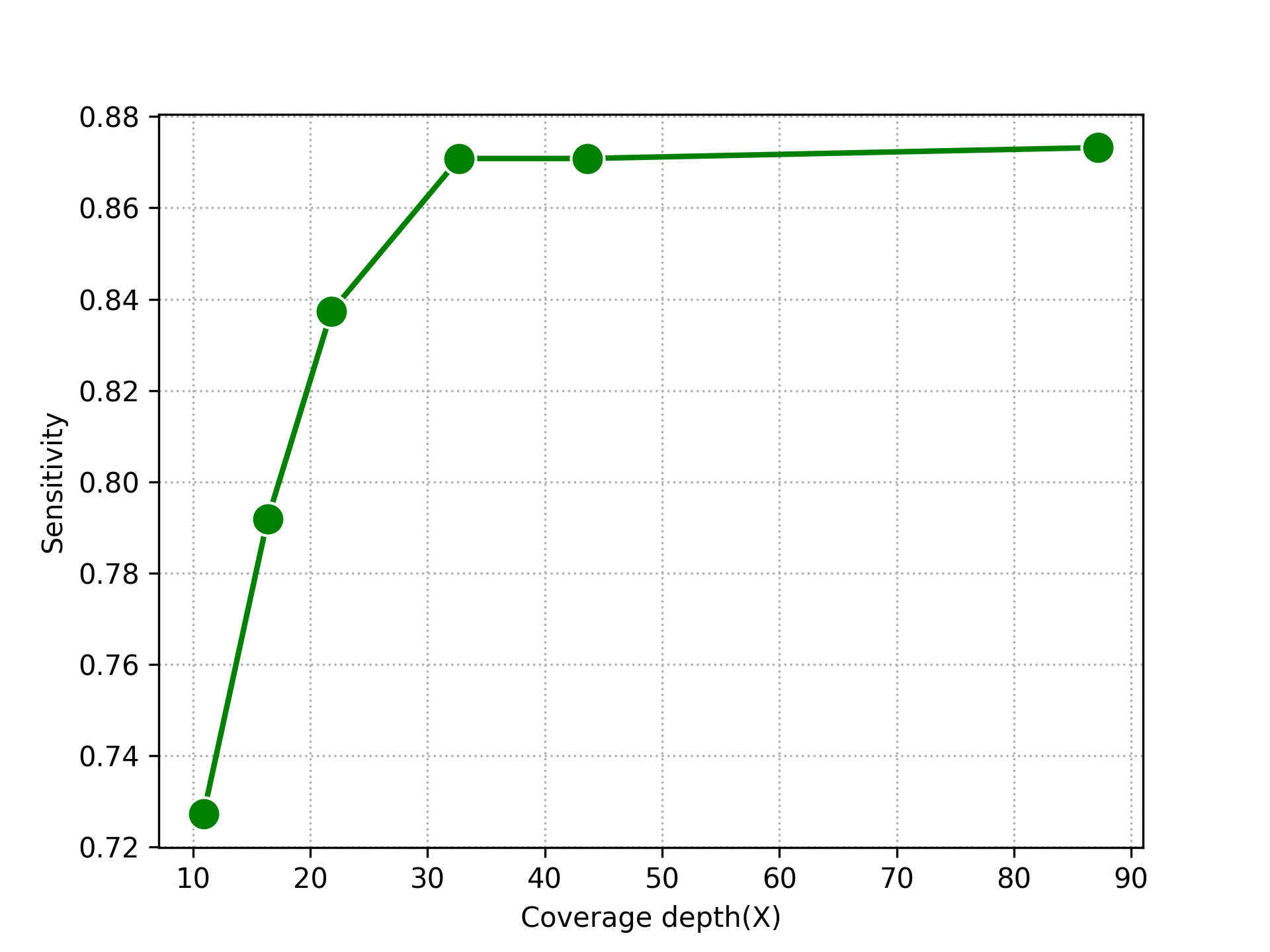
**

**Figure S3. Straglr’s genotyping performance versus sequencing depth.** Samples with different coverage depths were generated from random sub-sampling of the HG00733 PacBio sequences (SRR7615963). Sensitivity was calculated as the percentage of 418 heterozygous loci (see **Results**) with both alleles sized within 10% of the corresponding sizes determined from the diploid assembly.

**
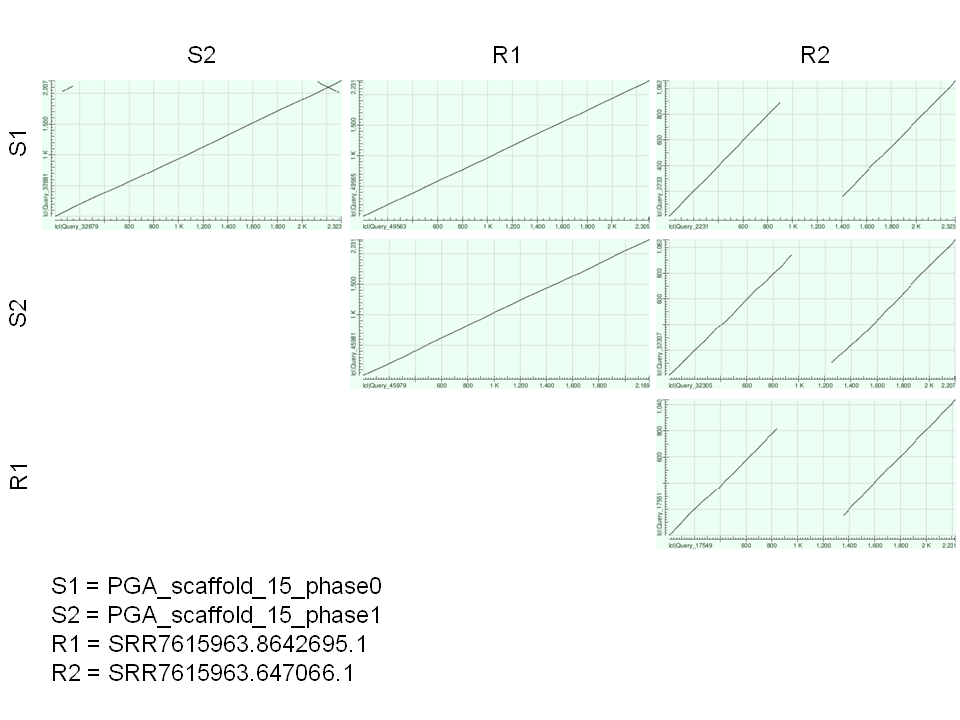
**

**Figure S4. Case of disagreement between assembly and Straglr genotype.** A homologous TR locus (chr4:12,648,301-12,648,903) in the HG00733 assembly identified as heterozygous with a smaller allele by Straglr. Sequences of the repeat with 200 bp flanks were extracted from the two scaffolds (S1, S2) in the assembly (2,007 and 2,123 bp), and two reads representative of the two alleles (R1, R2) reported in Straglr’s genotype (2,046 with 21 support reads and 872 bp with 36 support reads). The sequences were aligned against each other using the blast2seq web server (<https://blast.ncbi.nlm.nih.gov/Blast.cgi?PAGE_TYPE=BlastSearch&BLAST_SPEC=blast2seq&LINK_LOC=align2seq>) and the resulting DOT plots were captured. The smooth straight lines between S1 and S2, S1 and R1, and S2 and R1 indicate the same allele whereas the gaps between R2 and the other sequences indicate that it represents a shorter allele.

**
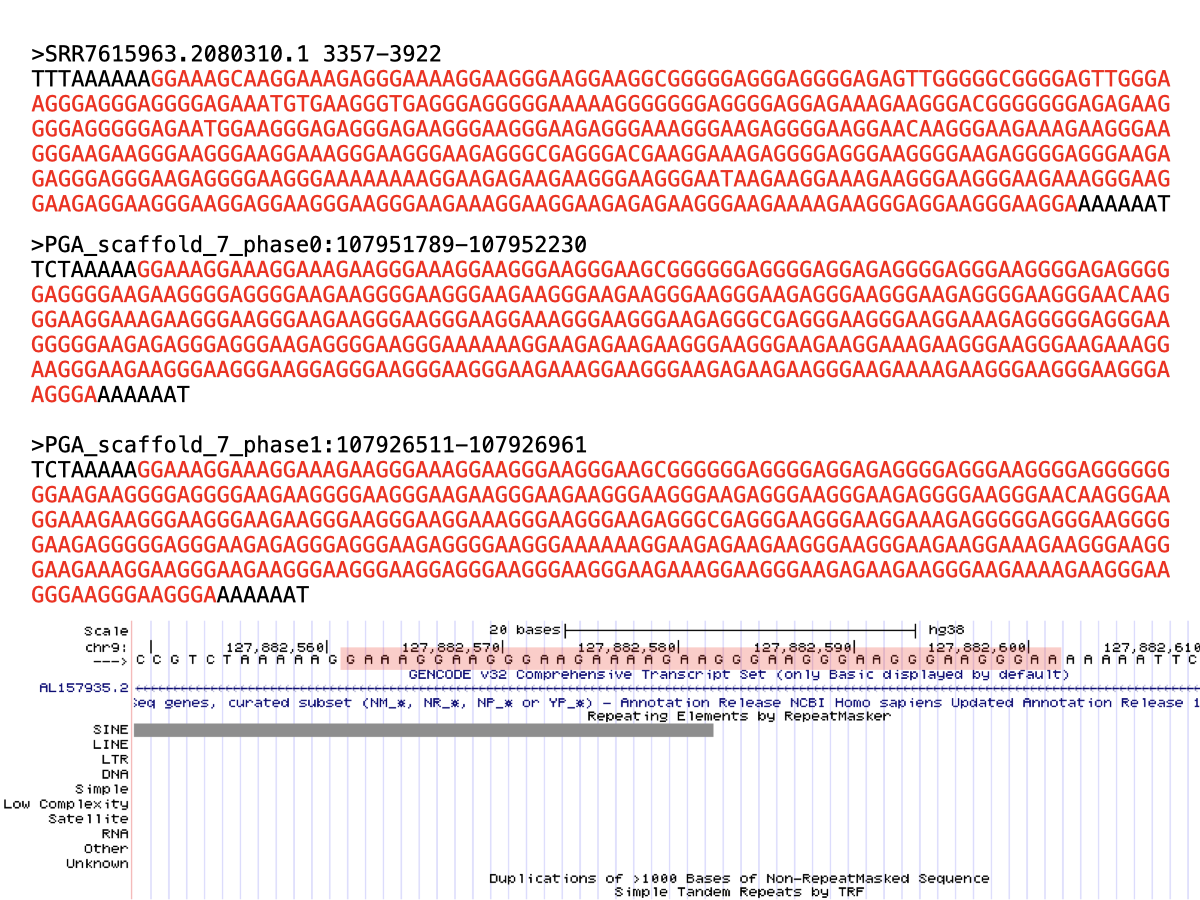
**

**Figure S5. Example of a “repeat expansion” in an unannotated locus in HG00733.** An example of a homozygous repeat expansion at unannotated locus (chr9:127,882,557-127,882,603) detected by Straglr’s genome scan in HG00733. The GAA repeat tract is highlighted in red for a representative supporting read, and the two assembly scaffolds. The UCSC genome browser track of the locus is shown at the bottom with the repeat sequence highlighted in red, and the empty Simple Tandem Repeats track at the bottom indicates that the repeat is unannotated.

**
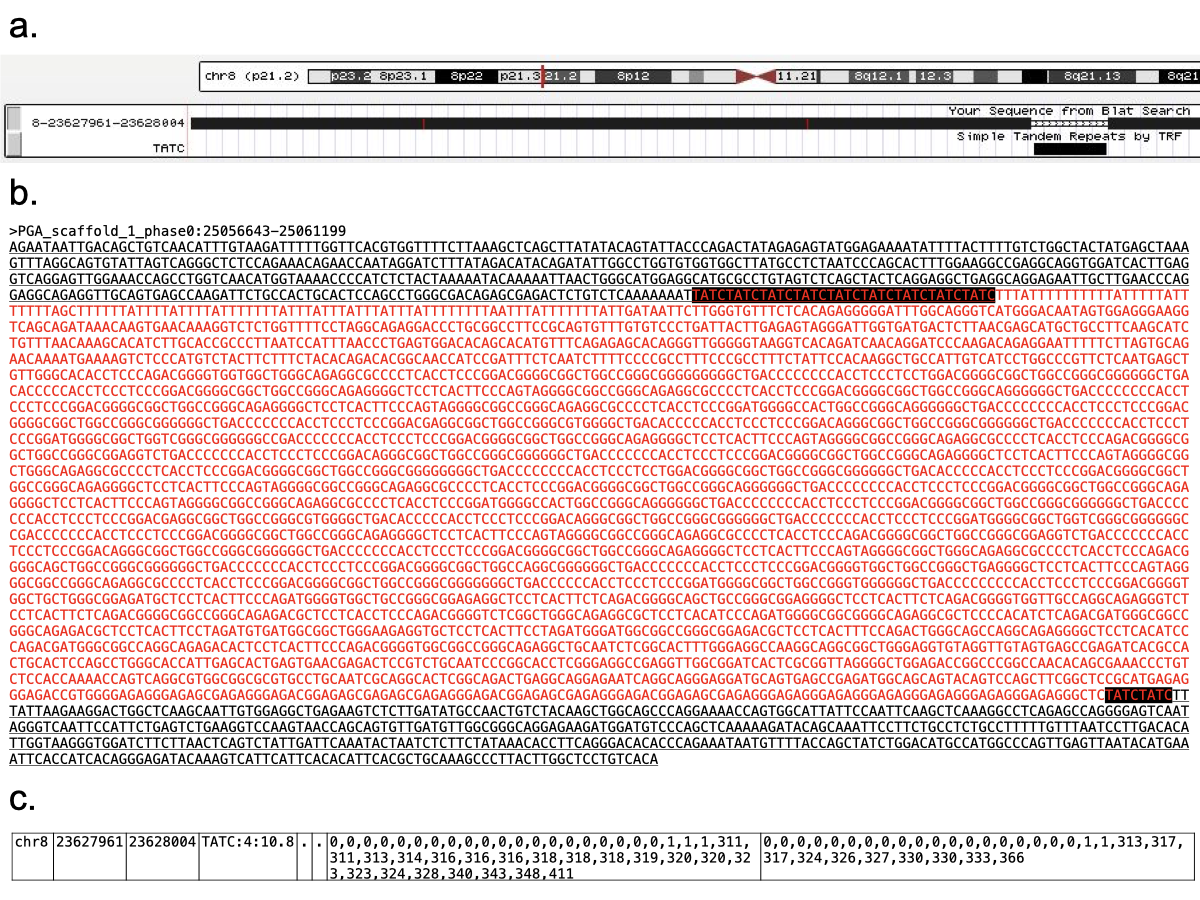
**

**Figure S6. Case of cryptic tandem repeat locus in HG00733 assembly.** An example case of cryptic tandem repeat locus in the HG00733 assembly **a.** UCSC genome browser track indicating the tandem repeat locus; **b.** One scaffold sequence of the allele reconstructed in the assembly. Bases with the black background indicate the annotated repeat (TATC) sequence. Bases in red and white background represent the 3.5kb insertion sequence **c.** Tandem-genotypes output of this target locus. The last two columns report the changes in the TATC repeat numbers relative to the reference genome for each supporting read on the forward and reverse strands respectively.
