# Supplementary material for: Straglr: discovering and genotyping tandem repeat expansions using whole genome long-read sequences
Source: Genome Biol. 2021 Aug 13;22:224. doi: 10.1186/s13059-021-02447-3 (PMC8361843; doi:10.1186/s13059-021-02447-3)
Supplement: Supplementary file 4 — Additional file 4. Review history. [file 13059_2021_2447_MOESM4_ESM.docx]

Review History

**First round of review**

**Reviewer 1**

**Were you able to assess all statistics in the manuscript, including the appropriateness of statistical tests used?** No, I do not feel adequately qualified to assess the statistics.

**Were you able to directly test the methods?** Yes.

**Comments to author:**

This manuscript by Chiu et al describes Straglr, a new software for analysis of tandem repeat elements from long-read sequencing data. Even though tandem repeats can be causative for severe genetic disorders, this is a class of genetic variation that often is overlooked. One of the main reasons for this is likely that tandem repeats are difficult to study with traditional genomics technologies. This has potential to change with long read sequencing. At present, few bioinformatics tools exist to detect tandem repeats from long-read sequencing data. In this context, Straglr could be an important addition to the toolbox, in particular since it relies on a standard alignment (minimap2) and could be easy to integrate into analysis pipelines.

The manuscript is well written and easy to follow. Benchmarking of Straglr was performed against Tandem-Genotypes and RepeatHMM, and this makes sense since these (in my knowledge) are the only other methods currently available. Despite that the performance of Straglr was rather similar to Tandem-Genotypes, I still find the method interesting mainly because it's a different implementation with potential benefits regarding computational performance and ease of use. At present it's difficult to know where this field is going and what tool is eventually going to become the gold standard for these types of analyses. Please see my comments and suggestions below:

Major points:

*     Simulated data is a big part of this study. This makes sense, since it's the most straight-forward way to evaluate the software. But one potential issue is that simulated data might not properly reflect the repeats that occur in human cells. Another issue is that all simulated data seems to be generated using NanoSIM, and reflects error profiles of ONT sequencing technology. It would be interesting to know the performance also on simulated PacBio HiFi data, but I suspect there is no way to generate such data. I would suggest that the authors discuss the challenges of using simulated data in the context of repeat elements and whether their results are valid for all long-read platforms. I also think the simulation results could be given a bit less space in the manuscript, and instead having more focus on the real sequencing data.

*     I found several of the figures difficult to digest, in particular Figs 1-4. In my opinion, they are too busy with lots of panels and colors. As a reader it's difficult to understand what is the take home message for each figure. My suggestion is to remove a lot of the information so each figure is clearly focused on the main message it is intended convey. The remaining information could be included in supplementary material, if it is at all necessary to display. Also, in my version of the manuscript, several of the figures have poor quality and are quite blurry. So please make sure to increase the resolution.

*     Related to the above point, I think the manuscript would benefit from simplification of the result text. In the current version, many numbers and percentages are presented throughout the results section. Please think again about which numbers feels relevant to highlight so that the reader is not confused by unnecessary information.

*     I tried installing and running the software on a local computer and on in-house generated data. The installation process was somewhat challenging, but I managed without too many problems. My aim was to analyze all TRs in the human genome in an in-house dataset, but don't think I managed to get through all the steps correctly since my output file was way smaller than expected. So as a user, I would find it valuable with a more detailed description on github on how to study all TRs in a human WGS dataset. Possibly similar information could be included also for other types of common analyses. Regarding the output file, I also noticed that each tandem repeat is represented as a separate line for each read it occurs in. It would be great if the output could (optionally) be collapsed into a genotyping result for each tandem repeat element.


Minor points:

*     The manuscript lacks page numbers, and this could be good to include in a future version.

*     On line 12 on the second page of Results, it's mentioned that Stranglr showed a p-value smaller than 0.05 for only the sample with expansion 500bp. Do you have any idea why this happens for this particular expansion size and not for smaller/larger sizes? Is it just by chance?
 
*     In the section on Targeted sequencing, I would like to see the exact repeat numbers for the No-Amp data. In Figure 4, the repeat distributions are plotted but no exact values are given for the peaks. Once such values have been obtained it will be easier to determine whether there are any differences in repeat expansion sizes for the different methods.

*     In the "WGS data" section in Results, it is mentioned that PacBio Sequel data has been analyzed. I think it would be good to be more specific here, since Sequel is just an instrument and doesn't say anything about what type of data was produced. I think it would be better to specify whether it is a continuous long read (CLR) or a HiFi dataset.

*     In the second paragraph of the "WGS data" section (lines 55-60), it is mentioned that Straglr and RepeatHMM have higher correlation coefficients (0.99) than tandem-genotypes (0.98). Is this really a significant difference or should you instead say that all methods had a similar performance?

*     In the last paragraph of the "WGS data" section (lines 26-34) a specific locus on chr9 is mentioned. Is this repeat located within a coding gene or not?

*     It could be interesting with a little more discussion on the coverage requirements for Stranglr. How much coverage is needed to get good results for a human WGS long read dataset? Do you need more coverage for ONT as compared to PacBio HiFi data?

**Reviewer 2**

**Were you able to assess all statistics in the manuscript, including the appropriateness of statistical tests used?** Yes, and I have assessed the statistics in my report.

**Were you able to directly test the methods?** No.

**Comments to author:**

The manuscript by Chiu et al. describes Straglr—software for targeted genotyping and novel expansion detection of short tandem repeats (STRs) from long-read sequences. The performance of the software was evaluated using simulations and comparing with two other STR typing tools. Straglr outperformed the other two tools. Straglr also has very high sensitivity and specificity. Next, the authors evaluated their tool by applying it to real targeted and whole-genome sequencing data. Straglr displayed high level of performance in this application. Overall Straglr is a useful tool for the research community and represents a significant advance over previous tools. I have several major concerns that the authors should address.

First, what is the lowest and the highest STR length that Straglr works on? Such information would be useful for future users. Relatedly, for shorter STR loci, what is Straglr's resolution in distinguishing sizes between the two alleles in a heterozygote? The real difference might be just 3 bp. Can Straglr detect it?

Second, for longer STR loci, what is the resolution in allele difference between alleles in a heterozygote? The authors mention that it is 100 bp for alleles in the 100 bp-1,000 bo range. This actually is not great, and approaches the resolution on an agarose gel. One expects a higher resolution from a sequencing experiment. Again, the real difference between the two alleles may be as small as 3 bp.

Does Straglr work to genotype and detect expansions for non-perfect repeats?

Beginning of Discussion:
- "Resolution limit increases to 200 bp or 300 bp". Should it be that "Resolution decreases"?

**Authors Response**

**Point-by-point responses to the reviewers’ comments:**

Reviewer 1 comments (major)

1. Simulated data is a big part of this study. This makes sense, since it's the most straight-forward way to evaluate the software. But one potential issue is that simulated data might not properly reflect the repeats that occur in human cells. Another issue is that all simulated data seems to be generated using NanoSIM, and reflects error profiles of ONT sequencing technology. It would be interesting to know the performance also on simulated PacBio HiFi data, but I suspect there is no way to generate such data. I would suggest that the authors discuss the challenges of using simulated data in the context of repeat elements and whether their results are valid for all long-read platforms. I also think the simulation results could be given a bit less space in the manuscript, and instead having more focus on the real sequencing data.

Authors’ response: We appreciate the reviewer’s concerns about the weight of simulation data used in the manuscript. We addressed the concerns by reducing the number of expansion sizes in the first simulation experiment from 9 to 3, and the number of base and separation sizes in the second experiment, such that the total number of combinations reduced from 30 to 8. We think this reduced set of allele sizes can still demonstrate the detection power and resolution of Straglr. We believe that the revised manuscript has achieved a better balance between simulated and real data.

We added a discussion about the limitations of simulation data, namely the difficulty in simulating repeat interruptions, complex events with different motifs or imperfect motifs. However, we highlighted how some of these challenges were addressed adequately with Straglr’s performance on real data from (a) targeted sequencing experiments for loci with multiple motifs; and (b) in WGS data, where loci of interest may have complicated/imperfect motifs.

As the reviewer speculated, to the best of our knowledge there isn’t any simulator for PacBio HiFi sequences to fit our purposes. PacBio HiFi data has higher accuracy over Nanopore or PacBio CLR sequences, and we think the performance of Straglr on such data can be inferred from its performance on targeted sequencing data.

2. I found several of the figures difficult to digest, in particular Figs 1-4. In my opinion, they are too busy with lots of panels and colors. As a reader it's difficult to understand what is the take home message for each figure. My suggestion is to remove a lot of the information so each figure is clearly focused on the main message it is intended convey. The remaining information could be included in supplementary material, if it is at all necessary to display. Also, in my version of the manuscript, several of the figures have poor quality and are quite blurry. So please make sure to increase the resolution.

Authors’ response: As mentioned above in our response to the viewer’s concern on simulations, we have cut down on the number of samples and hence the number panels in both figures 1 and 2. These should make the figures more legible. Fig. 3 is a single-panel figure that shows the resolution power and limits of Straglr, and is also presented in the revision. We also retained all the panels of Fig. 4, because it presents a comprehensive collection of No-Amp samples available, each with different repeat loci, different repeat sizes, and negative controls. We are open to moving the figure to the supplementary file if the reviewer thinks it makes for a smoother narrative.

We are sorry to hear that the production version of our manuscript had low-resolution figures, and apparently it continues to be the case. While the production processes of the journal are beyond our control, all figures are re-generated with bigger fonts and provided in TIFF format. The submitted figures are accessible through links in the combined production file, if a bit offset in the file that we received. We hope this is not too inconvenient for our reviewers.

3. Related to the above point, I think the manuscript would benefit from simplification of the result text. In the current version, many numbers and percentages are presented throughout the results section. Please think again about which numbers feels relevant to highlight so that the reader is not confused by unnecessary information.

Authors’ response: We noticed that the description of the genotyping evaluation results for both Straglr and RepeatHMM in the “WGS data” section is likely responsible for making the text a bit too “number-heavy”. We fixed this by replacing the original description laden with numbers and percentages with only sensitivity and specificity values for the two tools. The original numbers are now summarized in a table (Additional file 1 - Table S1). We believe that this change, together with the reduction of sample numbers in the first two simulation experiments, should make the new text more readable, while maintaining the message we hope to get across.

4. I tried installing and running the software on a local computer and on in-house generated data. The installation process was somewhat challenging, but I managed without too many problems. My aim was to analyze all TRs in the human genome in an in-house dataset, but don't think I managed to get through all the steps correctly since my output file was way smaller than expected. So as a user, I would find it valuable with a more detailed description on github on how to study all TRs in a human WGS dataset. Possibly similar information could be included also for other types of common analyses. Regarding the output file, I also noticed that each tandem repeat is represented as a separate line for each read it occurs in. It would be great if the output could (optionally) be collapsed into a genotyping result for each tandem repeat element.

Authors’ response: We are sorry to hear the difficulties the reviewer experienced in installing the software. The Python setup has been tested, and the pip command documented in the GitHub README should make the installation easier. Documentation on GitHub has also been improved with detailed step-by-step instructions provided for executing either a genome scan for larger-than-reference “expansions” or genome-wide genotyping.

Also we thank our reviewer for the feature request. We implemented a “locus-centric” format with all the relevant support read information collapsed into a single field per locus. This is executed by running with the new option “--simple” on the command line.

Reviewer 1 comments (minor)

1. The manuscript lacks page numbers, and this could be good to include in a future version.

Authors’ response: We thank our review for the suggestion. The revised submission has page numbers as requested.

2. On line 12 on the second page of Results, it's mentioned that Stranglr showed a p-value smaller than 0.05 for only the sample with expansion 500bp. Do you have any idea why this happens for this particular expansion size and not for smaller/larger sizes? Is it just by chance?

Authors’ response: We believe that out of all nine expansion sizes tested in the simulation experiment, only the 500bp case showing a KS test p-value below 0.05 is just by chance.

3. In the section on Targeted sequencing, I would like to see the exact repeat numbers for the No-Amp data. In Figure 4, the repeat distributions are plotted but no exact values are given for the peaks. Once such values have been obtained it will be easier to determine whether there are any differences in repeat expansion sizes for the different methods.

Authors’ response: The exact repeat sizes for every read in the targeted sequencing dataset both provided from PacBio analysis and determined by Straglr are provided in the new additional file (Additional file S3) under the columns “size_reported” and “size_st” respectively. The two showed a Pearson correlation coefficient of almost 1.00 from 15,793 reads with TR sizes ranging from 16 bpt to 7kb from the four target loci.

4. In the "WGS data" section in Results, it is mentioned that PacBio Sequel data has been analyzed. I think it would be good to be more specific here, since Sequel is just an instrument and doesn't say anything about what type of data was produced. I think it would be better to specify whether it is a continuous long read (CLR) or a HiFi dataset.

Authors’ response: We thank our reviewer for pointing out the need to clarify the sequence data type used in the WGS evaluation. We have added CLR to the description of the WGS dataset (line 179, page 8). This addition should also help distinguish the WGS from the HiFi dataset used in the targeted sequencing section.

5. In the second paragraph of the "WGS data" section (lines 55-60), it is mentioned that Straglr and RepeatHMM have higher correlation coefficients (0.99) than tandem-genotypes (0.98). Is this really a significant difference or should you instead say that all methods had a similar performance?

Authors’ response: We ran the Fisher Z-transformation to compare the two Pearson correlation coefficients, and the resulting p-val of 0 suggests the difference is statistically significant.

6. In the last paragraph of the "WGS data" section (lines 26-34) a specific locus on chr9 is mentioned. Is this repeat located within a coding gene or not?

Authors’ response: This locus resides in the intron of a coding gene AL157935.2 in the GENCODE v36 annotation. It appears that this gene may be a transcript variant of the neighboring ST6GALNAC6 gene as the first five exons are shared between the two genes in the annotation.

7. It could be interesting with a little more discussion on the coverage requirements for Stranglr. How much coverage is needed to get good results for a human WGS long read dataset? Do you need more coverage for ONT as compared to PacBio HiFi data?

Authors’ response: We conducted an experiment using various sub-samples of HG00733 data to examine the relationship between genotyping performance and the coverage depth. The samples were the same ones we generated for computing run-time assessment. Genotyping (accurate sizing of both heterozygous alleles) sensitivity of the 418 loci was used as the performance metric. We observed that genotyping performance increased with coverage depth, as expected, up to around 30X, after which the performance appeared to plateau. This result was added to the manuscript under WGS data in Results (lines 207-213, page 10), with the relationship plotted as Fig. S3 in additional file 1. The optimal coverage for WGS, approximately 30X as it appeared, was also mentioned in discussion (line 374, page 17). As PacBio’s HiFi sequences have better sequencing accuracy (over 99%) over Nanopore sequences, it is reasonable to assume less coverage may be sufficient for accurate genotyping.

Reviewer 2 comments

1. what is the lowest and the highest STR length that Straglr works on? Such information would be useful for future users. Relatedly, for shorter STR loci, what is Straglr's resolution in distinguishing sizes between the two alleles in a heterozygote? The real difference might be just 3 bp. Can Straglr detect it?

Author’s response: The lowest and highest STR lengths we have tested Straglr on are around 18 (reference FXN) and 9000 bp (simulation expansion). In response to reviewer’s question, we simulated 50 bp loci with heterozygous alleles separated by 60 bp and Straglr successfully distinguished the two alleles in 14/17 test loci. Alleles with slightly bigger separation of 70 bp were successfully distinguished in all 17 test loci. The resolution in genotyping heterozygotes by Straglr is obviously limited by the error rate of raw long-read sequences. Short STR heterozygotes, less than 100 bp in length, can be more reliably genotyped using short-read sequencing.

2. for longer STR loci, what is the resolution in allele difference between alleles in a heterozygote? The authors mention that it is 100 bp for alleles in the 100 bp-1,000 bo range. This actually is not great, and approaches the resolution on an agarose gel. One expects a higher resolution from a sequencing experiment. Again, the real difference between the two alleles may be as small as 3 bp.

Author’s response: In response to reviewer’s question, we simulated 500 and 1,000 bp STR loci (n=17) with alleles separated by 50, 60, and 70 bp. The result is as follows – numbers within the cells represent the number of loci with the two alleles distinguished:

| Base Allele\Separation | 50 | 60 | 70 |
| --- | --- | --- | --- |
| 500 | 8 | 13 | 17 |
| 1,000 | 6 | 8 | 10 |

The patterns observed here reflect the results reported and discussed in the manuscript – the confounding effect of indels in noisy raw long-reads tend to be larger for larger alleles with smaller separations and therefore resolution below 100 bp cannot always be achieved. This is, however, not a major concern as the main clinical application is to unambiguously differentiate normal, pre-mutation, and full-mutation alleles. The size difference between these clinically relevant classes is usually over 100 bp and can be confidently resolved by Straglr (see Mosaicism experiment in manuscript).

3. Does Straglr work to genotype and detect expansions for non-perfect repeats?

Author’s response: Yes, as the manuscript discussed, Straglr ran Tandem Repeat Finder with lenient parameters so that non-perfect repeats can be captured. In fact, many of the tandem repeats we used for benchmarking the HG00733 sample are complicated and non-perfect in the human genome. In addition, if specific interruption patterns are known, they can be specified along with the canonical pattern in the input bed file (comma-separated). In fact, this is how the target loci were specified for detection in the PacBio No-Amp dataset. Finally, in cases like polyglutamine diseases, the last degenerate base of the codon can be specified by an “*” in motif specification as an alternative to specifying the exact motif sequences.

4. Beginning of Discussion: "Resolution limit increases to 200 bp or 300 bp". Should it be that "Resolution decreases"?

Author’s response: This sentence is not in the new submission anymore.

**Second round of review**

**Reviewer 1**

The authors have addressed my comments.

**Reviewer 2**

The authors have adequately addressed my concerns.
